# A Practical Framework for Value Creation in Health Information Systems From an Ecosystem Perspective: Evaluated in the South African Context

**DOI:** 10.3389/fpsyg.2022.637883

**Published:** 2022-06-02

**Authors:** Sanelisiwe Hlongwane, Sara S. Grobbelaar

**Affiliations:** ^1^Department of Industrial Engineering, Faculty of Engineering, Stellenbosch University, Stellenbosch, South Africa; ^2^Department of Industrial Engineering, DSI-NRF Centre of Excellence in Scientometrics and Science, Technology and Innovation Policy, Stellenbosch University, Stellenbosch, South Africa

**Keywords:** ecosystem, information systems, value creation, stakeholder, healthcare

## Abstract

Performance improvement in any field depends on establishing goals that align the interests of relevant stakeholders, which may be defined as creating value for stakeholders. In the healthcare context, the concept of value creation and its analysis from an ecosystem perspective has been neglected and is hard to achieve in practice. This research adopts an innovation ecosystem perspective to develop and evaluate a practical framework to guide value creation for healthcare settings in a developing country context. The resulting framework serves as a tool that can guide stakeholders to co-create value by defining the inputs, activities, and outputs/outcomes to enable the process of value co-creation through a heath information system. Design Science Research Methodology (DSRM) was followed to develop the framework (artifact); it entailed the evaluation of the preliminary framework through a range of cycles. A relevance cycle was completed through a literature review. Since the investigation was done from an ecosystem perspective, it provided an understanding of the core characteristics of ecosystems, information systems, and value to inform the development of a preliminary framework. The preliminary framework was evaluated through two design cycles: the first was based on in-depth semi-structured interviews with six industry experts, and the second comprised a framework ranking exercise. The observations from the two stages informed the modification and refinement of framework items. The evaluated framework provides practical and actionable elements of a value creation system based on three canvasses: (1) the pre-use canvas defines the healthcare system and its stakeholders; (2) the tool guideline provides an overview of the development of ecosystem canvas elements; and (3) the ecosystem canvas represents the process of value creation along with a conceptual canvas with descriptions or implications of each of the framework’s concepts.

## Introduction

The emergence of healthcare as an important research area is attributed to the critical role of healthcare in modern socio-economic development (Lee et al., 2015). Investing in healthcare contributes to a country’s economic growth, improved living conditions, and social infrastructures (Assamala, 2014). In particular, Health Information Systems (HIS) are considered as key investments to address rising challenges, and are fundamental in the delivery of healthcare (Lee et al., 2015; Sligo et al., 2017).

The healthcare industry, compared with other industries, is lagging in the adoption of formal strategies for information systems planning. This is partly due to the complexity of the healthcare system and, in South Africa, to disparate legacy systems that are difficult to integrate. In addition to recognizing the crucial issue of strategic planning, it is also imperative to elucidate the impact of value co-creation in the success of health information systems (Al-yaseen et al., 2010).

Improving performance and accountability in any field is dependent on establishing goals that unite the interests of all stakeholders. This goal could be defined as: “to create value for stakeholders.” In healthcare, value encompasses and integrates many of the already existing goals within the healthcare system such as quality, safety, patient centricity, and cost management, which bring together the interests of actors such as patients, payers, providers, and suppliers (Porter, 2010; Kupfer and Bond, 2012; McColl-Kennedy et al., 2012).

Value co-creation is the process by which value is generated through interactions between multiple stakeholder groups (Thomas and Autio, 2012; Hardyman et al., 2015). Ecosystems provide a means of analyzing dynamic and massively interconnected organizations, technologies, and actors through a holistic and multi-actor lens (Anggraeni et al., 2007). Understanding multiple stakeholder ecosystems and how the process of value creation takes place is an important enabler of a holistic view of the system (Pinho et al., 2014). It allows for a focus on the whole complex ecosystem to gain a deeper understanding of where and how value emerges from the collaboration of ecosystem actors.

While a thriving body of literature exists on the development of ecosystems and the process of value capture (Khademi, 2020) it mostly explores business and private sector domains, with some studies focusing on data-based value through big data (Grover et al., 2018; Lim et al., 2018), business models, and business performance (Di et al., 2021). Further, a recent review highlighted the importance of considering ecosystem actors at a micro level to obtain a holistic understanding of ecosystem and how it functions. In addition, it is important to understand that ecosystem formation does not necessarily lead to value creation, only the opportunity to do so; the latter remains largely dependent on how participants behave and pursue opportunities through value co-creation (Hlongwane and Grobbelaar, 2020). The literature review further indicated that there are no papers with a specific focus on Africa. The void of relevant literature substantiated the need to gain insight into the challenges facing digital systems and their ability to create value in the South African context. Therefore, while a focus on value systems and co-creation from an ecosystem perspective is a growing area of research, the development and evaluation of grounded frameworks and models in an African context remains scarce (Ketonen-Oksi and Valkokari, 2019; Laubscher and Saville, 2021) [see section “Literature Review (Part 1 – Relevance and Rigor Cycle)” for more information].

From a contextual point of view, South Africa’s (SA) history of discrimination of individuals based on race and gender has profoundly affected its health policies and services (Coovadia et al., 2009). Post 1994, the ruling African National Congress (ANC) aimed to address the disempowerment, discrimination, and underdevelopment that characterized the delivery of healthcare services (Coovadia et al., 2009). The public healthcare system was made the cornerstone of health policy and the intention was to transform the healthcare system into an integrated and comprehensive national service that would allow all people access to essential healthcare (Coovadia et al., 2009). Despite breakthroughs achieved through post-1994 innovations, their success has been restricted by the failure to delegate authority and by the erosion of efficiencies due to factors such as lack of leadership, corruption, low staff morale, and financial constraints (Harrison, 2009; Ratshidi et al., 2020; Spies et al., 2020). South Africa is thus still grappling with massive healthcare inequalities (Coovadia et al., 2009; Marten et al., 2014). Evidence shows that the COVID-19 pandemic has exacerbated this situation by driving further inequalities in access to healthcare services, and in particular community healthcare (Okoi and Bwawa, 2020; Nwosu and Oyenubi, 2021).

This motivates the development of an HIS management tool to ensure long-lasting economic and environmental sustainability in healthcare by considering the roles, mechanisms, and individual actors that form part of the healthcare system.

The study addressed the following main research question: *“What constitutes a practical framework to guide the development of value creation processes in information systems in the South African healthcare ecosystem?”*

The South African case illustrates that numerous challenges affect a healthcare system’s ability to deliver value to its stakeholders in an efficient and effective manner through health information systems. The research objective is to develop a guideline and tool to explore various co-creation practices to generate value for all stakeholders and approaches in developing an HIS. We take an ecosystem perspective to this problem.

To develop successful HISs, literature has shown (1) the importance of a clear vision and a shared value base; and (2) the facilitation of engagement by ecosystem actors to engage and make connection and diversity to drive value co-creation processes (Ketonen-Oksi and Valkokari, 2019).

This article develops and evaluates the utility of a practical framework in developing HISs in a developing country context:

1. The practical framework must assist in defining the relevant healthcare ecosystem and its stakeholders by identifying the requirements and considerations that need to be noted for successful co-creation of value for the HIS; and
2. The practical framework must outline how to define the inputs, activities, and output/outcomes to enable development of a clear implementation strategy to enable value co-creation in the healthcare ecosystem when developing the HIS.

A Design Science Research (DSR) methodology was adopted, as motivated and mapped to the layout of this article in section “Methodology.” Section “Literature Review (Part 1 – Relevance and Rigor Cycle)” outlines the literature review, section “The Preliminary Framework (Part 2)” presents a preliminary framework (artifact), section “Results: Framework Evaluation in the South African Context (Part 3 – Design cycles)” presents the results, and section “Discussion (Part 4)” presents the evaluated framework (artifact), discusses the findings, and reflects on managerial implications and future work.

## Methodology

“Design science research is a “lens” or set of synthetic and analytical techniques and perspectives” (Hevner and Chatterjee, 2010: 1). DSR is widely used in the area of information systems, health care, education, engineering, and computer science to create new or expand existing knowledge and improve current practices by creating artifacts and analyzing the use or performance thereof through iterative evaluations and reflections (Hevner, 2007). DSR – sometimes referred to as improvement science – is used to address complex real-world problems that occur in complex settings and involve various stakeholders (Dreschler and Hevner, 2016).

DSR applies to Socio-Technical Systems such as IS and is often used in IS research (Hevner et al., 2004). DSR “seeks to create innovations that define ideas, practices, technical capabilities, and products through which the analysis, design, implementation, management, and use of IS can be effectively and efficiently accomplished” (Hevner et al., 2004: 76).

The size and complexity of the problems and solution spaces when applying DSR in IS means that it is not always possible to identify an optimal solution. Instead, DSR focuses on discovering satisfactory solutions that suffice the solution space without explicitly specifying all possible solutions. The design involves creating, utilizing, and assessing heuristic search strategies and emphasizing that the solution works in context. The iterative process of DSR makes it possible to simplify the complex problem into smaller subsystems and then improve the satisfactory solution or expand the scope of interest with each iteration (Gregor and Hevner, 2013). It is therefore considered as a suitable approach to this research study.

The goal of this study is to develop a management framework and tool for assisting developers of health information systems to consider value creation from an ecosystem perspective. We followed the Design Science Research Methodology (DSRM), as proposed by Peffers et al. (2008), to develop the management framework (artifact). This consisted of six activities: (1) problem identification and motivation; (2) defining solution objectives; (3) design and development; (4) demonstration; (5) evaluation; and (6) communication (Hevner and Chatterjee, 2010).

Design Science Research as defined by Hevner and Chatterjee (2010) requires that knowledge and understanding of the design problem and solutions be acquired throughout the process of building and applying the artifact. The final outcome of DSR is to deliver an evaluated artifact that creates knowledge about the design problem and the solution; it is tested and developed throughout the DSR process and therefore has utility to users (Hevner and Chatterjee, 2010).

Hevner (2007) makes the point that DSR is dependent on a process that integrates a series of cycles in the development of an artifact, namely, (1) the relevance cycle; (2) the design cycle; and (3) the rigor cycle.

**FIGURE 1:**
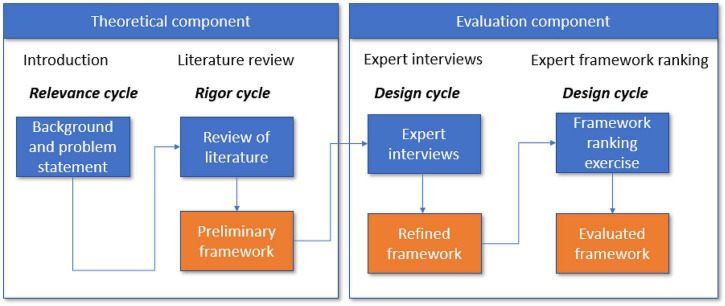
Overview of theDSR process followed in this research.

As shown in Figure 1, the relevance cycle triggers the research and helps to formulate the problem statement and the framework requirements (see section “Introduction”) (Hevner, 2007; Dreschler and Hevner, 2016). This is an important phase of the work, as the level to which the development of the artifact is appropriate, applicable, and implementable needs to be ensured to fit its implementation environment (Hevner, 2007).

The rigor cycle refers to the development of legitimacy to ensure that the artifact is grounded in knowledge by drawing on the knowledge base. This may include exploring existing frameworks, theories, models, and instruments that may be used in the development or evaluation of the research artifact (Dreschler and Hevner, 2016; Scribante et al., 2019). Part 2 (Theoretical component) of the study presents the knowledge base from published literature and explores the problem landscape. Section “Literature Review (Part 1 – Relevance and Rigor Cycle)” presents the outcome of a literature review to obtain an overview of the multidisciplinary literature related to value, information systems, and ecosystems. The outcome of section “Literature Review (Part 1 – Relevance and Rigor Cycle)” inspired the development of the subsequent preliminary framework through a rigor cycle as prescribed in the DSR process [discussed in section “The Preliminary Framework (Part 2)”].

The design cycle involves the rapid, iterative construction, and evaluation of the artifact that draws from both the real-world environment and knowledge bases (Hevner and Chatterjee, 2010; Hevner, 2007). Dreschler and Hevner (2016) note that the evaluation of the artifact could be done in an artificial setting (e.g., conceptual applications) or directly through an application. Part 3 comprises a process, discussed in section “Results: Framework Evaluation in the South African Context (Part 3 – Design cycles),” that iteratively refined and evaluated the framework. This was achieved through semi-structured interviews with industry experts to evaluate the concepts in the framework and to gain additional insight. A framework-ranking exercise was used to evaluate the relevance and utility of various aspects and dimensions of the framework. The findings and results of the evaluation process are discussed in section “Results: Framework Evaluation in the South African Context (Part 3 – Design cycles).” The communication activity of the DSR process is contained in section “Discussion (Part 4),” which presents the final framework and tool.

With reference to Figure 1, the article is organized in three major sections. The detailed methods followed in Parts 1 to 3 are presented in sections “Part 1: Outcome of a Literature Review (Rigor Cycle),” “Part 2: Preliminary Framework Development (First Development of an Artifact),” “Part 3: Framework Evaluation: Methods for the Interviews and Framework Ranking Exercise (Design Cycles to Refine the Artifact),” and “Part 4: Discussion.”

### Part 1: Outcome of a Literature Review (Rigor Cycle)

The review and identification of core concepts in section “Ecosystems as Concept for Value Creation and Value Capture” presents the fundamental concepts identified in the literature review, and illuminates the interpretation by this study of value logic, stakeholder symbiosis, and institutional stability. It links the ecosystem literature with HIS.

The selected papers were critically appraised. This process involved identifying the main attributes, characteristics, and assumptions from the papers and then categorizing the concepts based on their ontological, epistemological, and methodological roles. The outcomes of the literature review are discussed in section “Literature Review (Part 1 – Relevance and Rigor Cycle),” specifically to show the synthesis framework that could be developed from the core concepts identified.

### Part 2: Preliminary Framework Development (First Development of an Artifact)

Based on the understanding of the main concepts derived in Part 1, this stage of the investigation provided an understanding of the core characteristics of ecosystems, information systems, and value, which in turn informed the development of the preliminary framework, along with the inventory of important concepts identified through the literature process.

### Part 3: Framework Evaluation: Methods for the Interviews and Framework Ranking Exercise (Design Cycles to Refine the Artifact)

The first stage of the evaluation process included semi-structured interviews with industry experts, who were selected based on a snowball sampling process. The process of saturation was applied to determine the number of interviews that were conducted. Although the sample is small, it is regarded as sufficient to arrive at a more refined framework through various steps and iterations.

The purpose of the interviews was to gain insight from three perspectives, namely, (1) researcher; (2) developer; and (3) healthcare perspectives. Interviewees were selected based on their expertise in value creation, ecosystem management, governance, health national standards, and health information systems. The designations, qualifications, and reason for the inclusion of each participant is presented in Table 1. The first four interviewees formed part of the interviewee process, and later participated in the framework ranking process together with the remaining three participants.

**TABLE 1:** Interviewee and framework ranking participants.

#	Designation	Qualification	Reason for inclusion
1	Chief researcher (CSIR)	Ph.D. in information technology	They are established researchers who have a vast background in healthcare research, and who also have contributed to the development of the National Health Normative Standards Framework.
2	Chief researcher (CSIR)	Ph.D.	
**3**	Lead Solutions Engineer	Masters in Medicine, Biomedical Engineering BSc Electronic Engineering	Interviewee was selected based on rich industry experience of over 29 years, role at Jembi, and past academic history in the healthcare field.
4	Healthcare practitioner	MBChB	Interviewed as an expert practitioner to gain insight into healthcare complexities and health information systems.
5	Healthcare practitioner	MBChB	Contributed as an expert practitioner. This facilitated insight into healthcare complexities.
6	Healthcare practitioner	MBChB	Contributed as an expert practitioner. This facilitated insight into healthcare complexities.
7	Product Manager	Master’s degree in Engineering/Industrial Management	To gain insight into digital healthcare solutions

The interview questions were categorized into the six development parts: (1) governance; (2) co-creation; (3) information and knowledge sharing; (4) external environment; (5) organizations/institutions; and (6) stakeholders. This simplified the structure of the data that were transcribed, as the data gathered from the interviews were easily divided into one of the six parts (see section “Interview Discussion Guidelines for Semi-Structured Interviews” in Supplementary Material for detailed interview questions).

**FIGURE 2:**
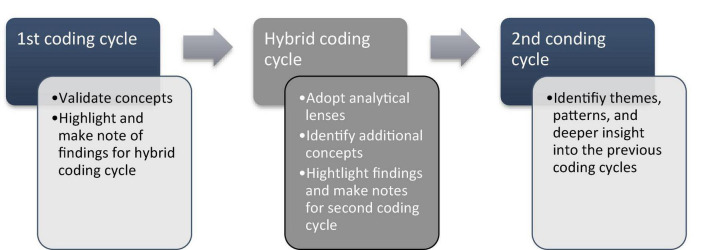
Coding cycles of interview data (Saldaña, 2013).

Following the interviews, Creswell’s approach for analyzing and interpreting data were used to make sense of the data gathered (see Figure 2). Creswell’s approach suggests segmenting the data into smaller parts for investigation and putting it back together again. The first cycle focused on determining whether the interviews validated the concepts included in the framework based on the perspectives and worldviews of the interviewees. A second, hybrid cycle was incorporated to ensure that the data were sufficiently analyzed. The final cycle yielded refined data that consisted of themes, patterns, and deeper insight into the relationships and links between the data.

The second stage of the evaluation process consisted of a framework ranking exercise. The framework ranking exercise, which used the framework ranking sheet presented in Supplementary Table 6 in the Supplementary Material, provided an opportunity to confirm the transferability of the framework, given its development from multiple literature sources that spanned across multiple disciplines and varying developed countries. The valuable observations from the two activities led to the modification and refinement of the framework items, which resulted in the evaluated framework [Presented in section “Discussion (Part 4)”].

### Part 4: Discussion

The interviews and examination of the interview data transformed the one-dimensional framework into a three-dimensional framework consisting of canvases specific to the South African health context (see Part 4). This part of the article presents the evaluated framework, followed by a conclusion section that outlines managerial implications and shortcomings of the research (see section “Conclusion”).

## Literature Review (Part 1 – Relevance and Rigor Cycle)

### Ecosystems as Concept for Value Creation and Value Capture

This study adopts and ecosystem perspective that has become progressively common in both research and in practice. It draws on the concept of natural ecosystems to provide a way of looking at a business’ structure, interactions, and exchanges, and achieves this by shifting the analysis of a business network to the systems level by focusing on the relations, interactions, and dynamics of massively interconnected organizations, technologies, and actors (Anggraeni et al., 2007). The attractiveness of the approach and the driving force behind selecting the ecosystem perspective for this study lies in its ability to provide a lens that focuses on self-organization, coevolution, adaption, and co-creation of value (Peltoniemi and Vuori, 2004; Thomas and Autio, 2012).

The “ecosystem” term has grown in its ecological meaning and has raised awareness of new models of value creation and value capture (Aarikka-Stenroos and Ritala, 2017; Adner, 2017). Two views that have enabled conceptualization of these models in the ecosystem context include: ecosystem-as-affiliation and ecosystem-as-structure. The ecosystem-as-affiliation realm is a strategy that views ecosystems as a community of interconnected actors, technologies, and institutions that are defined by their network and platform affiliations (Aarikka-Stenroos and Ritala, 2017; Adner, 2017). The strategy offers an appealing metaphor that is helpful for the description of interactions and links between actors at the macro level. However, the ecosystem-as-affiliation perspective is limited in its ability to provide a comprehensive understanding of value creation. This is mainly due to its focus on general governance and community enhancements. The alternative perspective, the ecosystem-as-structure, offers an approach that considers interdependent value creation. The approach starts with a value proposition that is linked to a business model that focuses on achieving sustainable development and offering long-term solutions to for multiple stakeholders (Bocken et al., 2014). The approach obtains a constellation of stakeholders that need to interact in order for the value proposition to come to a realization (Adner, 2017; Jacobides et al., 2018).

Three key defining characteristics of an ecosystem provide a framework to better understand ecosystems and also serve to set the boundaries for the ecosystem construct (Thomas and Autio, 2012). The first characteristic is the importance of the *value logic*, in particular the source of value and how it is created. The second characteristic is the *symbiotic relations* of stakeholders in the ecosystem, as each stakeholder provides specialized and complementary inputs for value creation and co-evolve to maintain the stability and health of the ecosystem. The last characteristic is the *institutional stability* within an ecosystem, in which a locus of coordination is established to provide structure for the operation of governance mechanisms that coordinate the ecosystem (Thomas and Autio, 2012; Anomah and Agyabeng, 2013). These concepts are briefly introduced and defined in the sections below.

#### Defining “Value Logic”

Several attempts have been made to create a holistic conceptualization of value, which include defining value as: (1) the amount that a consumer is willing to pay for a firm’s offerings; and (2) the properties of the products or services that provide benefits to the consumer (Grönroos and Voima, 2013; Garriga, 2014). These conceptualizations are traditional ideologies of value and are grounded in the conventions and models of an industrial economy. The concept of value has grown to include new ideologies that consider the value creating system itself. Here different actors such as suppliers, customers, and business partners work together to co-produce value (Grönroos and Voima, 2013; Garriga, 2014). This ideology suggests understanding the boundaries of value logic by including the notion of the source of value and value co-creation, as these are key elements of the construct (Saarijärvi et al., 2013).

Different forms of value emerge for different actors through different processes when it comes to “value,” “co,” and “creation” (Saarijärvi et al., 2013). The difference lies therein that value creation refers to a consumer’s creation of value-in-use, where value emerges for the user during a goods or service activity; however, value co-creation is a function of interactions between ecosystem actors (Alves et al., 2016). Successful value co-creation requires ecosystem actors to be able to interact with one another through the exchange and integration of resources within the context of their own reality (Breidbach and Maglio, 2016).

#### Defining “Stakeholder Symbiosis”

Literature proposes a narrow and instrumental definition of stakeholders as a group of individuals without whose support the organization would cease to exist (Reed et al., 2009). Broader and more normative definitions also exist that view stakeholders as entities that are affected by the performance of the organization (Reed et al., 2009). This study considers a combined definition that views the stakeholder(s) as *“any group or individual who can affect or is affected by the achievement of the organization’s objectives”* (Reed et al., 2009). This definition was further adapted by replacing “achievement of the organization’s objectives” with “creating, maintaining, or extending a symbiosis” (Hein et al., 2017). This is owed to the fact that the idea that stakeholders are interdependent and have the ability to forge symbiotic relationships, and therefore have a “stake” in a symbiosis (Hein et al., 2017), is central to most interpretations of stakeholder theory. The symbiosis concept is essential to explore due to its collaborative properties that allows for traditionally separate actors to collaborate for the purpose of gaining a competitive advantage (Gibson, 2012).

The symbiotic relationship between stakeholders in an ecosystem builds upon the notion of co-evolution, which is considered as a joint outcome of both co-specialization and complementariness in an ecosystem (Ekanayake et al., 2017). From the co-evolution perspective, ecosystems are shaped by stakeholders who continuously act and react to the environmental changes and pressures that arise as a result of other stakeholders (Verdu et al., 2012). In this regard, ecosystems evolve by means of mutual influences, which are the inputs that facilitate value co-creation. Co-specialization emanates from the need to support the ecosystem and therefore drive its performance by providing specialized inputs. From the stakeholder’s perspective, co-specialization enables each stakeholder to contribute their core capabilities through collaboration in order to drive the ability to create value. Interactions are an important dimension that is necessary to ensure the success of co-specialization and therefore the realization of value creation. It is expressed through the functional characteristics of each stakeholder, as well as through their responsibility in the ecosystem (Thomas and Autio, 2012).

#### Defining “Institutional Stability”

From the ecosystem perspective, emphasis is placed on the central role of actor-generated institutions and institutional arrangements that influence the trajectory of institutional stability and change (Verdu et al., 2012; Siltaloppi et al., 2016). This perspective suggests that actors are embedded in a set of interrelated rules and norms that encompass coordination, legitimacy and trust, and governance mechanisms. Here, actors can jointly reconstruct and change value co-creation practices to allow for new solutions to emerge, which ultimately advance change in the institutional arrangement. This is vital for the creation, development, health, and maintenance of an ecosystem (Thomas and Autio, 2012; Siltaloppi et al., 2016). Institutional theory provides a useful lens to understand the organizing principles, rules, and norms in ecosystems. The three institutional characteristics of Institutional stability include: *coordination*, *legitimacy and reputation*, and *governance mechanisms.*

Ecosystem coordination drives the network’s performance by enabling both value creation and sharing. A critical element of coordination is the underlying architecture that connects all participating actors (Jacobides et al., 2018). This underlying architecture forms the central actor that coordinates the ecosystem, which is vital for its health and stability (Thomas and Autio, 2012).

*Legitimacy and reputation* provide the validity that organizations seek in their decision to participate and remain in an ecosystem (Stoll et al., 2010). These aspects are vital for its survival and to ensure that the ecosystem is greater than the sum of its parts. Through active management of reputations and relationships, the uncertainty, ambiguity, and conflict among ecosystem participants can be minimized (Thomas and Autio, 2012).

The *governance structure* is perhaps the most salient aspect of an ecosystem (Sharapov et al., 2013). These mechanisms exercise power and authority in ecosystems by instilling conventions such as rules and norms to govern the behavior of participants in the ecosystem (Sharapov et al., 2013). Accordingly, governance is an important mechanism that orchestrates and manages the manner of communication between different parties (Thomas and Autio, 2012). For an ecosystem to be successful and for its robustness not to be threatened, participants must conform to the values, rules, and norms shared within the ecosystem (Thomas and Autio, 2012; Sharapov et al., 2013).

### Mapping the Concepts: Synthesis From the Literature Review

**FIGURE 3:**
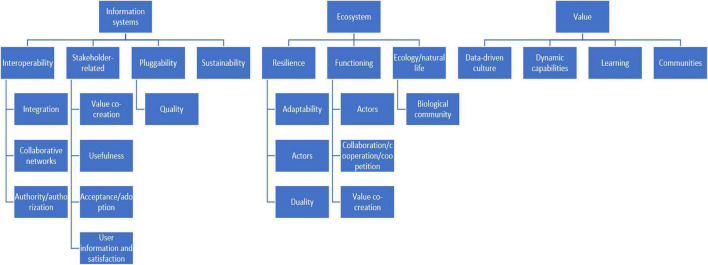
Synthesis of concepts from the literature review.

Following the search strategy and methodology discussed in section “Part 1: Outcome of a Literature Review (Rigor Cycle),” the review was conducted to develop an initial synthesis of the landscape of value creation and information systems research from an ecosystems perspective. The investigation of the diverse ecosystem literature led to the identification of important and frequently emerging concepts relating to value, information systems, and ecosystems (see Figure 3). These concepts are presented in Supplementary Table 4 in the Supplementary Material. A brief reflection on the initial concepts included in the framework follows below.

#### Information Systems Concepts Synthesis

Information systems introduce new ways to combine and exchange resources to create value for the actors in the exchange (Barrett et al., 2015). Information systems need standards enforced by a regulating body to integrate dissimilar systems and to support interactions across networks regardless of the physical and operating systems (Serbanati et al., 2011; Schiza et al., 2019). Supplementary Table 4 in the Supplementary Material outlines four subcategories related to information systems, namely, interoperability, sustainability, pluggability, and stakeholder-related concepts. Interoperability relates to ability to share and make use of information (Salih Zeki et al., 2011). Sustainability relates to developing innovative digital data-based designs that transform businesses and drive economic development, leading to greater efficiency and cost reduction (Barrett et al., 2015; Pappas et al., 2018). Pluggability refers to incorporating quality standards that are a reflection of the external quality criteria for information technology services. These standards are equivalent to reliability, efficiency, and/or maintainability (Aulkemeier et al., 2016).

The last subcategory for information systems relates to stakeholder-related concepts. These concepts are key, since information systems are used by interconnected actors. The value created by information systems, and therefore its success, is largely dependent on the behavior, capabilities, and needs of the stakeholders; thus, it is important to ensure that the information needs and requirements of the stakeholders are satisfied (Pappas et al., 2018).

#### Ecosystem Concepts Synthesis

The ecosystem concept yields fundamental aspects to be considered regarding how information systems function from a holistic perspective. The first subcategory includes concepts that influence the resilience of the ecosystem such as adaptability, actors, and duality. It is important to acknowledge that different actors function at different stages with the system. This ultimately affects the systems adaptability when disturbance in the system occurs (Kharrazi et al., 2016). The second subcategory is the functioning category, which focuses on evolving the way in which ecosystem actors interact, cooperate, and collaborate to create value (Barrett et al., 2015; Pappas et al., 2018; Heim et al., 2019). The final subcategory, ecology, focuses on biological community, and considers the interaction between entities with their environment. Ecology also draws from the business ecosystem literature.

#### Value Concepts Synthesis

According to the primary studies, there are several theoretical concepts that need to be considered with respect to value. These concepts include data-driven culture, dynamic capabilities, learning, and communities. In a data-driven culture, value is created by extracting data that have purpose and meaning in giving actionable insight and allowing actors to base their decisions on insight instead of instinct (Pappas et al., 2018; Joda et al., 2019). These actors are actively integrated with varying needs and capabilities in order to foster collaboration and a bond through competences, relationships, information, and a shared vision (Osório et al., 2010; Barrett et al., 2015; Adler-Milstein et al., 2017; Pappas et al., 2018). The evolving perceptions and needs of the actors should be continuously monitored and studied to increase this value (Tarafdar and Tanriverdi, 2018).

### Structural Component of Studies for Supporting the Relevance Cycle

The unit of analysis of the entities studied in each of the ecosystems varied. These entities were grouped into three broad categories, and included: the political and economic environment, the organization, and the primary stakeholders.

These factors play an important role in defining requirements as per the DSR process, where the environment and the requirements of the artifact are explored.

#### External Environment

The literature indicated that the environment may be unpredictable due to political, economic, and social instability (Primmer et al., 2015). The external environment has been found to form pre-existing conditions that either provide new opportunities for value creation or hinder the success of the value creation system (Mainardes et al., 2012). This is largely because these factors may act as constraints that shape the environmental structure (Barrett et al., 2015). The strategic behavior of the organization is subject to these factors, with the organization needing to respond in accordance with their respective importance (Primmer et al., 2015). Due to the impact that the external environment has on the organization, it was considered that its role in the value creation process could be of importance to encourage flexibility and adaptability in changing circumstances that may arise (Medema et al., 2017).

#### The Organization

The organization, which is termed the “bridging organization” in the framework, is recognized in literature as a key feature for collaboration, as it forms an intermediary between the diverse stakeholders and their networks in support of the value creation process (Barrett et al., 2015). The main purpose of the bridging organization is to facilitate the development of a network that brings together multiple positions, knowledge types, and sources while providing a platform for value creation (Medema et al., 2017). The idea is for these collaborative networks to become learning networks that cultivate continuous value co-creation, improvement of practices, and institutional development. The bridging organization therefore provides an environment for new collaborative networks to arise for the purpose of developing new social practices and interactions (Medema et al., 2017).

The literature indicated that deliberate co-creation processes, enabled by the organization, may be necessary to facilitate a neutral space for open and iterative dialog so as to allow stakeholders to learn and share knowledge for the purpose of co-constructing new, innovative, and personalized experiences (Medema et al., 2017). Though co-creation is considered to be the center of gravity in the design of organizational services, literature suggests a shift from the inside of the organization to its environment for the purpose of stimulating innovativeness (Adamik et al., 2018). A deeper understanding of the environmental factors of the organization’s networks may be necessary to understand their impact on the organization’s desired outcomes. This is important as these networks are dynamic in nature and continuously changing (Adamik et al., 2018). Further, these political, cultural, and institutional factors play a large role in the power and therefore information asymmetries within the organization, which in turn influences the organization’s co-creation process (Adamik et al., 2018).

Information sharing through the use of information systems is a notable concept that emerged from the literature, as it is said to be essential to the survival of an organization in the environment (Panetto et al., 2016). Information systems form an integral part of efficient and effective information sharing within an organization. Literature suggests that seamless interfaces to facilitate sharing of vital information may be needed to perform varying functions using the same set of resources (Medema et al., 2017). Information sharing therefore encourages the distribution of useful information for systems, people and organizational units (Panetto et al., 2016). Repeated interactions through information sharing have the potential to build strong network ties between network members, which could eventually lead to high levels of trust. It also leads to the development of a shared understanding, vision, purpose, and culture (Lotfi et al., 2013). Literature emphasizes the contribution of information sharing to the success of the value creation process, and its role in the value creation system is therefore considered. This success includes how information sharing: (1) reduces costs; (2) improves relationships with stakeholders; (3) increases the flow of resources; (4) enables efficient delivery of services; and (5) facilitates the achievement of a competitive advantage (Medema et al., 2017). To attain this success, the information systems that facilitate information sharing must have semantic interoperable capabilities that support collaboration across platforms (Salih Zeki et al., 2011). Semantic interoperability goes beyond merely sharing information, and deals with its interpretation to ensure that the transmitted information is fully understood by the receiver (Panetto et al., 2016).

#### The Stakeholder

Evidence from literature has shown that the power of stakeholder networks lies in their diversity, which may lead to a more robust value creation system (Panetto et al., 2016). The network refers to a set of relationships that connect the participating stakeholders to one another. Elements from governance mechanisms, namely, hierarchical governance, scientific-technical governance, adaptive collaborative governance, and the governance of strategic behavior are used to characterize the stakeholder network in the framework. This is mainly due to the findings that suggest that these governance modes influence how the ecosystem functions by taking into account the people and the organization’s decision-making processes (Thomas and Autio, 2012; Primmer et al., 2015).

The consideration of readiness together with the ability of stakeholders to engage in value co-creation practices also emerged as important (Medema et al., 2017). It was found that this aspect naturally encourages stakeholders to form symbiotic relationships that allow for traditionally separate stakeholders to engage in the value co-creation process (Medema et al., 2017). To successfully facilitate the co-creation process and therefore create value, it is suggested that stakeholders may need to be jointly involved in the process to ensure value formation. However, value formation is not necessarily guaranteed, as the process can be either creative or destructive. The quality of the interactions between stakeholders are fundamental to successfully create value, as is the organization’s understanding of the stakeholder outside of the value creation process (Hein et al., 2017). Understanding and learning more about the stakeholder and their individual context and how that influences the value creation process aid in the effective management of these interactions (Grönroos and Voima, 2013).

#### Outcomes of Value Creation Processes

Understanding the desired outcomes of the value creation process may be needed to determine the necessary activities to be performed by the organization. These outcomes are dependent on the credibility, salience, and legitimacy of the value creation efforts (Medema et al., 2017). For the value creation process to be considered credible, the collaborative stakeholder network should deliver timely and useful outputs; These include synthesized feedback meetings and reports that discuss the rigorous measurement of the value created. This essentially allows for: (1) ongoing reflection on the effectiveness of the value creation process and its outcomes; (2) the discussion of lessons learnt; and (3) driving systemic progress (Grönroos and Voima, 2013; Medema et al., 2017). Legitimacy refers to the extent to which the value creation efforts acknowledge the sources of value, which differ for different participating stakeholders in the ecosystem (Medema et al., 2017). Flexibility, efficiency, and innovation form the unique sources of value that govern and henceforth act as drivers of the legitimacy of the value creation system (Medema et al., 2017). The salience of the value creation process refers to the quality of the knowledge that is used, modified, and shared within the value creation system (Thomas and Autio, 2012).

### The Requirements for the Artifact

A key practice in designing a solution through DSR is developing a sound relevance cycle outcome. The design outline essentially provides an idea of the intended solution prior to the development of the fully detailed design, and involves formulating key requirements that are needed to guide the design process. Van Aken and Berends (2018) use categories to group the different requirements that should be addressed by a design. These include:

1. Functional and structural requirements: key specifications that usually relate to the performance or the demands of the designed solution;
2. Boundary conditions: the design requirements that need to be met and cannot be negotiated; and
3. User requirements: the requirements relating to the use of the framework.

The research draws inspiration from these categories to develop the design requirements of the framework. The latter were deduced from the literature and are linked to the strategic categories discussed in section “Literature Review (Part 1 – Relevance and Rigor Cycle).” The set of requirements to be met by the framework needed to meet are summarized in Table 2.

**TABLE 2:** Artifact requirements deduced from literature.

Framework requirements	Code	Description and reference in research study
Functional requirements	FR1	The framework should identify how collaborative environments can be formed within a healthcare system
	FR2	The framework should provide fundamental value creation activities needed within a healthcare system
	FR3	The framework should highlight the role of information systems in value creation within the ecosystem
	FR4	The framework should encourage the evolution of healthcare systems through interactions, cooperation, and collaboration
	FR5	The framework should acknowledge the different governance modes that influence how the ecosystem functions
	FR6	The framework should encourage transparency through free and unrestricted sharing of up-to-date and useful information and knowledge
	FR7	The framework should provide an understanding of how stakeholder groups can effectively support knowledge co-creation by including components that either hinder or provide opportunities for collaborative stakeholder networks
	FR8	The framework should show how traditional components of co-creation can be utilized in complex and ever-changing environments
Structural requirements	SR1	The framework should address the theoretical underpinnings of the dynamic ecosystem construct and its actors
	SR2	The framework should adopt a holistic system perspective to conceptualize the ecosystem construct by considering the three health system levels, which include: the political and economic environment of the health system, the healthcare facility, and the primary stakeholders
	SR3	The framework should encourage active integration and collaboration of stakeholders with varying needs and capabilities to increase value
	SR4	The framework should address the network of explicit and implicit relationships that span both the internal and external environment
Boundary requirements	BR1	The framework should reflect the boundaries within which value is created in a healthcare system enabled by information systems
	BR2	The framework should assist ecosystem actors, who share the same institutional logic, with a set of common rules and norms to govern their behavior in the ecosystem
	BR3	The framework should support value co-creation through networked relationships
User requirements	UR1	The framework should assist users with tools to address complex challenges affecting value creation
	UR2	The framework should assist users to understand how value can emerge through information systems by providing them with favorable actions for value creation
	UR3	The framework must assist users in understanding the dynamics of the ecosystem and the implications thereof

## The Preliminary Framework (Part 2)

**FIGURE 4:**
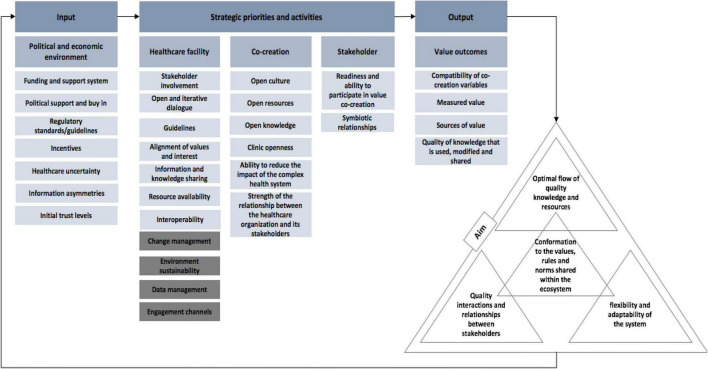
Preliminary framework.

The preliminary framework of the value creation system aimed to include the factors that could address the complexities within healthcare systems, and further aimed to determine how value could emerge from the collaboration between participants who interact using HISs. Trends and key elements were used as building blocks to formulate the preliminary inventory framework for the interpretation of a value creation process in healthcare ecosystems (see Figure 4). This framework identifies the main factors that may be considered in improving the value creation process that is supported by information systems in a healthcare ecosystem. A more detailed account of the various factors is provided in Supplementary Table 5 in the Supplementary Material.

The integrated structural components and functions of the value creation system (as defined by the systems requirement) provide a view of different units of analysis and groups of stakeholders that can guide the strategy development process. This organization of structural concepts provides a perspective that encourages the consideration of the three health system levels, which include: the political and economic environment of the health system, the healthcare facility, and primary stakeholders. These three levels shape the healthcare system and were used in the development of the framework to explore their inter-relatedness.

The functional components of the ecosystem perspective highlight the co-creation aspect, such as readiness to co-create, appropriate resources for the co-creation process, knowledge sharing, and reducing the complexity of interaction between stakeholders, technology systems, and the facility/organization structure. The value of strengthening relationships and networks is seen as central to achieving this. Governance is a core aspect of achieving institutional stability; it relies on the institutions and institutional arrangements and needs to acknowledge the different governance modes that influence ecosystem functioning.

Value outcomes consider factors that influence the desired outcomes of the value creation process and reflect the findings that were incorporated into the framework due to their role and significance in the value creation process.

## Results: Framework Evaluation in the South African Context (Part 3 – Design Cycles)

The DSR evaluation process and design cycles comprised semi-structured expert interviews to evaluate the framework. The results from the evaluation process informed the progressive modification of the framework, resulting in the refined framework and management tool (artifact).

### Results From Semi-Structured Interviews

The reflection on findings from the expert interviews (1) identified additional concepts to incorporate into the framework and (2) highlighted areas of disagreement. These results are presented in Table 3.

**TABLE 3:** Results of the semi-structured interviews.

Development part	Validated concepts	Additional insights	Disagreements	Additional concepts
External environment	Politics and economics have a great impact on the outcomes of the healthcare system, as does political buy-in.	The effect that corruption has on access and affordability of healthcare, efficiency, policy, and healthcare expenditure.	In public healthcare, only the patients, healthcare workers, and government are allowed to play a role. No external investment is allowed.	Impact of corruption on the health system. The impact of healthcare reform.
	There are standards that the health system needs to adhere to and aim to achieve.			
	Interoperability standards need to be adhered to in support of use of health information systems and to facilitate secure and seamless exchange of information.	Consideration of the role of decentralization in the healthcare system.		
Organization	Resources need to be available to ensure that the healthcare goals are met.	Creating sustainable value in healthcare within the context in which it exists.		Creating sustainable value that sustains the healthcare system.
	Health system interactions between the entities and the rest of the ecosystem.			
	There needs to be a consideration of the different sources of value.			
	Development of a structured approach for the adoption and implementation of changes.			
	Systems used in healthcare need to adhere to the standards and guidelines put in place, otherwise they will not be of value to the healthcare organization.			
	Consideration of value-in-context, which conceptualizes the dynamics of value within multidimensional networks.			
Stakeholder	Value is created for the beneficiaries (stakeholders).	The development of sustainable value propositions for each stakeholder group.		Development of sustainable value propositions for stakeholder groups
	A collaborative approach toward stakeholders successfully achieving shared goals and creating value within the healthcare system.			The influence that the value creation process has on stakeholder satisfaction.
	The involvement of patients in the decision-making process is important, as they are the most important people in the value chain.			
Co-creation	Transparency facilitates co-creation between stakeholders in the healthcare system.	It is important to co-create with marginalized communities, especially the illiterate and uninformed people.	Value is co-created at different scales; there is no instance where there is a single creation of information. Information by nature is co-created.	Stakeholder characteristics and their role in the willingness of stakeholders to participate in the co-creation process.
	Co-creation plays a role in an organization’s ability to adapt to changes in a relatively fast manner.	Consideration of the social dimension in co-creation, particularly with regard to differing cultures and different languages.		The consideration of social and human capital
	Co-creation brings in multiple perspectives to ensure that the process of co-creation is successful and of value by getting more than one perspective.			The influence of adverse attitudes of healthcare officials toward participation of certain stakeholder groups.
Information and knowledge sharing	Information sharing through information systems streamlines the health system, which is especially important in terms of service delivery.	In order for health information systems to function successfully, they need to be affordable, easy to use, and easy to implement.	People who use information systems to share information are not always the people who get value from it. The people who actually get value out of these systems are up-stream. The value experienced gets more and more the further away one gets from the point of use.	The impact of silos on interoperability challenges, communication barriers, and disjointedness of the healthcare organization.
	Information systems are not currently playing a very large or effective role in the public health sector due to their poor implementation.	Data silos significantly contribute to interoperability challenges.		Infrastructures that form the foundation for information and knowledge exchange
	There is no value in information that cannot be shared.	Data quality is essential for the effective use of information systems.		The crucial role of data quality for the effective use of information systems and influence on information value.
	Information and knowledge sharing improves communication and aids in the effective management of healthcare practices, resource allocation, and resource flow.	Information evolves and new information may emerge over time, and it needs to be reviewed to assess its significance before making any new changes.		
	Using information and knowledge to identify opportunities for value creation.			
	Sharing information and knowledge is necessary to get different perspectives in order to create value.			
				

Certain topics and concepts, which were continuously mentioned and discussed throughout the interviews, were identified as trends and patterns following application of the four analytical lenses. These trends and patterns were considered in the design and development of the value creation system in the South African healthcare context.

The first trend/pattern is governance and its role in the healthcare system. Various standards and guidelines were designed to manage the functions, activities, processes, and structures of the healthcare system and its components. Involvement of relevant stakeholders in the decision-making processes and development of these standards and guidelines broadens the consensus on the most appropriate strategy for success. While development of standards is important, the crux of their importance lies in their ease and effective implementation to ensure that the desired goals and objectives are reached.

The next set of trends relates to information systems, and included information and knowledge sharing, interoperability and standards, value of information and the adoption of information systems. Information and knowledge sharing are essential for decision making, healthcare improvement, value creation, and identifying value opportunities. Lack of information and knowledge sharing can be detrimental and affect the success of the healthcare system. Interoperability and standards play a crucial role in information sharing to harness the value of information and knowledge by providing a fundamental linkage and integration of information and knowledge in a way that enriches healthcare data. The value of information and knowledge that is used and shared through these systems increases when it is accurate, reliable, and up to date. To further harness the value of information, information systems must be stable. This means that information systems must encompass resilience in the face of disturbances that transcend the scope of known properties to ensure that that system does not fail or lose information. There is value in ensuring that information systems are adaptable in such a way that people can adopt it. This is achieved through simplicity, autonomy, localization, ease of use, and ease of implementation.

The following set of trends relates to the co-creation of value creation in healthcare. The aim of co-creation differs between interacting stakeholders, as stakeholders have different agendas and objectives with the co-creation process. While co-creation is for some intended to improve systems, processes, and the overall experience and satisfaction of the patient, others may co-create for economic purposes. This can result in individuals behaving purely for the benefit of their own interest rather than for that of the collective. The aim of co-creation also varies with the level at which co-creation takes place. Co-creation can scale from the healthcare provider and patient levels to healthcare workers co-creating one electronic health record, which in turn can contribute to co-creation at the provincial and country levels. A variety of factors influence the co-creation process and its success, and can be viewed as either obstacles to, or supporters of, the process. In this sense, these factors are considered to be “two sides of the same coin.”

The final two trends relate to healthcare and to stakeholders. Both these trends have a significant influence on the design, development, and implementation of the value creation system. The notion of the healthcare organization and what it encompasses needs to be emphasized as healthcare differs in scope and level. This is especially important in the South African healthcare context that requires the consideration of varying constraints and complexities. This will further assist in the identification of the relevant stakeholders that need to be considered as stakeholders vary in healthcare environments.

### Results From Framework Ranking Exercise

A framework ranking exercise was conducted to explore the relevance and usefulness of the framework. The outcomes from framework ranking were used to gain insight into the importance and implementation difficulty of various framework items.

**FIGURE 5:**
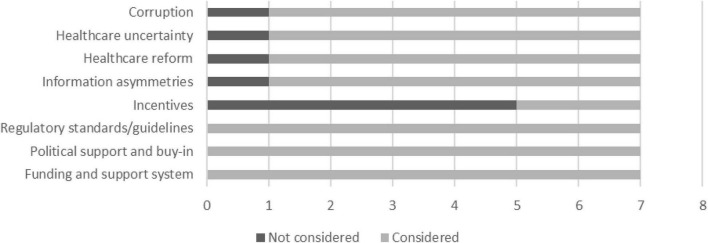
Consideration of external influencing concepts.

**FIGURE 6:**
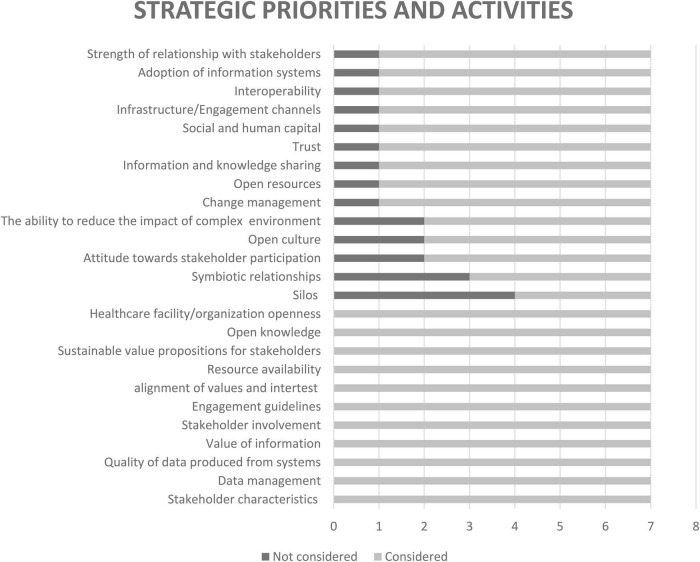
Consideration of concepts used to strategically prioritize and conduct activities.

**FIGURE 7:**
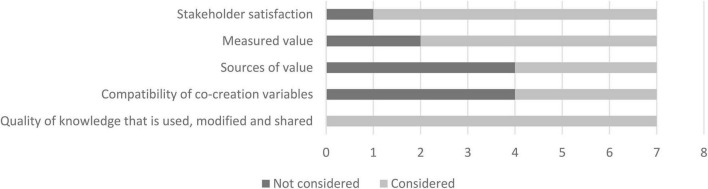
Consideration of concepts that address desired value outcomes.

An analysis of the data collected during the ranking exercise is discussed in this section. Feedback regarding the consideration of the framework’s concepts in the applied world is presented in Figures 5–7. From the graphs, it is clear that the majority of the framework’s concepts were considered by all participants, with only 15 concepts classified as not considered by some participants. Here, Incentives ranked the highest overall as the concept that is not considered in the design, development, and implementation of digital interventions. This is followed by Silos, Symbiotic relationships, Sources of value, and Compatibility of co-creation variables. It was deduced from the notes provided by the participants and from further enquiry that lack of knowledge, limited resources, and the nature of some of the concepts in given instances contribute to why some concepts are not considered.

**FIGURE 8:**
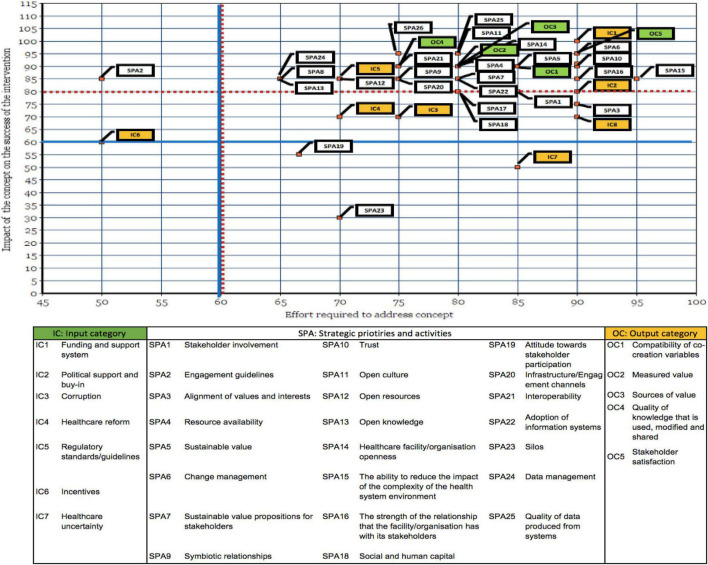
Comparison of the respective degrees of impact and effort for each concept Discussion (Part 4).

A primary motivation behind the framework ranking exercise was for the researcher to identify the impact and effort required to address the framework’s concepts from a collective group of industry experts. Figure 8 presented at the end of this section, maps the impact of the respective concept on the success of a digital intervention against the effort required to address the concept. The graph compares the cumulative frequency at which the respective degrees of impact and effort was selected by the participants for each concept. These data were subsequently useful as an indication of what experts regard as priorities. This was done by identifying concepts that were deemed to have a positive or an extremely positive impact, but that require a moderate, high, or extremely high degree of effort to address or implement.

In Figure 8, each concept was coded using a label and a color that represents the category under which it falls. Indicators that are deemed as input [input category (IC)] are represented by the yellow blocks in the graph; strategic priorities and activities (SPA) are shown in white; and indicators that may be seen as outputs [Output category (OC)] are presented in green.

In Figure 8, concepts that have a positive or an extremely positive impact with a moderate, high, or extremely high degree of effort need to be prioritized in a value creation system. This “cut off” is indicated by the red dotted lines, and these concepts fall above and to the right of these lines, as well as on the red dotted lines. Four concepts, labelled IC3 (Corruption), IC4 (Healthcare reform), IC8 (Information asymmetries), and SPA3 (Alignment of values and interests) fall slightly below the red dotted line but still above the blue line. This means that these concepts have a moderate to positive impact with a high to an extremely high degree of effort needed to address or implement. These four concepts also present the need to be prioritized due to their positions on the graph. This decision is further supported by Figures 5, 6, as these concepts are considered in the applied world by majority of the participants. The final concept that needs to be prioritized is SPA2, as this concept is deemed to have a positive impact with a minor degree of effort needed to address it. The selection of this concept is also supported by Figure 6, which shows the concept ranked as considered by all industry experts.

The ranking exercise enabled the researcher to identify concepts that were deemed to have no impact, a negative impact, or an extremely negative impact, but require a moderate, high, or extremely high degree of effort. These concepts include IC6 (incentives), IC7 (healthcare uncertainty), SPA19 (attitude toward stakeholder participation), and SPA23 (silos). The discussion of this outcome, together with the investigation into the prioritization of IC6, IC7, and SPA19, is beyond the scope of this study. In regard to SPA3, the insight gained through interviews suggests that data silos significantly contribute to interoperability challenges and therefore need to be addressed. This notion is also confirmed by Reda et al. (2018).

## Discussion (Part 4)

### Evaluated Framework

The proposed management tool consists of three overarching dimensions, each with their own canvases. (1) The first dimension, the pre-use canvas, supports definition of the healthcare system and its stakeholders by highlighting the requirements and considerations to be noted prior to the use of the tool. (2) Dimension two forms the tool guideline, which gives an overview of the development parts of the ecosystem canvas. These development parts were formulated with the South African healthcare context in mind. (3) The final and third dimension forms the ecosystem canvas, which represents the process of value creation in the healthcare context. This canvas is accompanied by an additional conceptual canvas that provides the descriptions or implications of each of the framework’s concepts to complete it.

The dimensions and their canvases characterize important strategic features of a value creation system that have been considered in a healthcare ecosystem. These dimensions are intended to assist researchers, policymakers, and health care workers to understand how a value creation system, which is supported by information systems, can be used to address and possibly overcome challenges faced within a healthcare organization. The final dimensions and their canvases are discussed in the sections that follow, and are presented in a legible size at the end of the chapter.

#### Dimension One: The Pre-use Canvas

**FIGURE 9:**
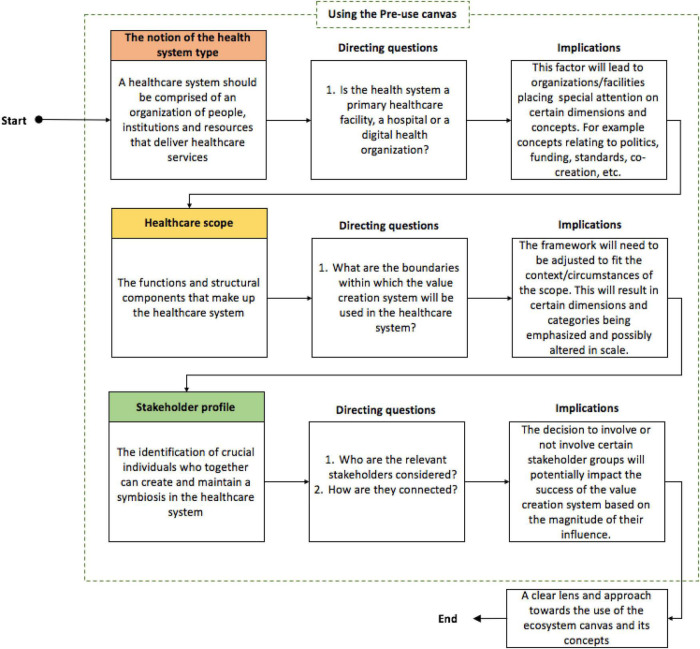
The pre-use canvas of the final management tool.

Healthcare is not an activity that has one type of action; hence, setting a perspective to narrow the scope is important. Throughout the process of evaluating the framework, the researcher realized the importance of clearly defining the healthcare profile as it greatly influences the lens used to view the framework. The Pre-use canvas, presented in Figure 9, highlights the importance of establishing the healthcare profile. Here, the notion of the healthcare system, healthcare scope, and stakeholder profile are the three factors comprising the healthcare profile that were found to influence the approach toward the framework. These factors need to be established prior to the use of the framework.

There is value in starting the value creation process with a clearly defined healthcare profile. This is important, as there are implications that need to be considered for each of the components comprising the healthcare profile when using the framework. The notion of the healthcare system type is used to establish the unit of analysis and whether the healthcare system is a primary healthcare facility, hospital, or digital health organization. The framework was developed to be as generalized as possible, thereby allowing it to be utilized in these varying healthcare system types. Defining the healthcare system type is important as it results in an emphasis on certain framework items. The healthcare scope forms the second component that is used to define the healthcare profile. Here, the scope of the healthcare system under which the framework is used needs to be defined. This is essential, as the framework needs to be adjusted to fit the context or the circumstance of the scope, which in turn will place further emphasis on certain framework items.

Following the first two components of the healthcare profile is the consideration of the stakeholder profile. Stakeholders have the potential and ability to affect the success of a healthcare system. This is largely based on the magnitude of the influence that they have, which varies from stakeholder to stakeholder. Defining the stakeholder profile is therefore necessary to determine which relevant stakeholders are considered. The range of stakeholders involved in a healthcare system forms the foundation for value creation and co-creation. Therefore, the decision to involve or not involve certain stakeholders has the potential to impact the success of the value creation system.

#### Dimension Two: The Tool Guideline

The second dimension of the framework is the tool guideline, which has two overarching aims, the first of which is to facilitate the design, development, and implementation of a value creation strategy used within a healthcare ecosystem. The tool guideline aims to achieve this by guiding the user through the typical development parts that form the dynamic building blocks of a successful value creation system. The second aim is to educate users by providing them with a branch of knowledge on the various development parts that form the foundation for value creation in a healthcare system. Here, the users are informed about the practical and actionable elements of a value creation system that draws from the literature review and interviews.

**FIGURE 10:**
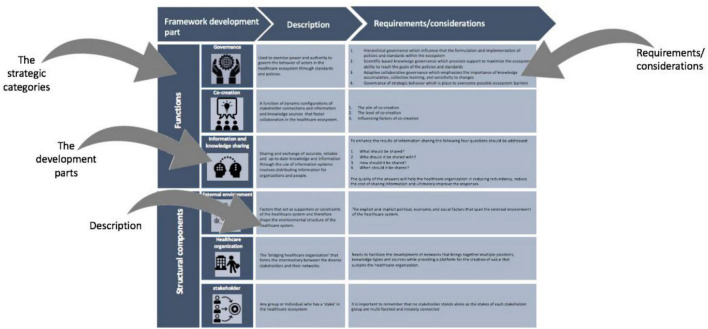
The tool guideline of the final management tool.

The tool guideline, of which the structure is presented in Figure 10, was developed with the South African healthcare context in mind. The layout of the tool guideline includes the six development parts that form the functions and structural components of the value creation system. The figure clarifies the terminology used in the discussion of the tool guideline, and highlights the possible actions that are required or should be considered within each development stage.

The tool guideline presents the six development parts of the value creation system, namely: (1) governance; (2) co-creation; (3) information and knowledge sharing; (4) external environment; (5) healthcare organization; and (6) stakeholders. These development parts form the functions and structural components of the value creation system and are grouped accordingly. Governance, co-creation, and information and knowledge sharing are classified as the functions of the value creation system. The remaining development parts, namely the external environment, healthcare organization, and stakeholders are classified as the structural components of the value creation system.

Governance forms the first development part, classified as a function of the value creation system. Governance refers to the actions and rules used to govern the healthcare system and considers the people and the organization’s decision-making processes. Governance influences how the ecosystem functions and is therefore key for the success of the healthcare system. The second function of the value creation system is co-creation. Co-creation elucidates the importance of fostering collaboration between healthcare system actors as a neutral space for open and iterative dialog. Co-creation essentially allows stakeholders to learn and share knowledge between one another to attain personal and institutional capacity for the purpose of co-constructing new and innovative solutions in an efficient manner. The last development part, classified as a function of the value creation system, is information and knowledge sharing. This development part considers the management and use of information and knowledge to support healthcare processes in creating value. Here, the “what,” “who,” “how,” and “when” information should be shared is considered. Sharing information and knowledge encourages co-creation, and by facilitating the sharing of information, through governance and standards, the care that people receive improves.

The external environment forms the first development part, classified as a structural component of the value creation system, and refers to the external influences that shape the strategic behavior of a healthcare system. These influences form the pre-existing conditions that either hinder or provide new opportunities to create value. For this reason, the external environment plays a vital role in the structure of the healthcare system. The healthcare organization forms the second structural component of the value creation system, and is recognized as a key feature that is necessary to foster a collaborative environment between diverse stakeholders within their networks. In this sense, the healthcare organization forms an intermediary between these stakeholders, which encourages co-creation and therefore value creation. The final structural component of the value creation system is the stakeholder development part; this refers to the group of individuals whose “stake” and influence has a great impact on the success of the value creation system. Stakeholders play an important role in the healthcare ecosystem as they shape the ecosystem by continuously acting and reacting to environmental changes and pressures that arise because of other stakeholders and additional influencing factors.

The structural components, together with the previously discussed functions, are arranged to form Dimension three of the management tool (see section “Dimension Three: The Ecosystem Canvas”). It is important to note that the governance, information and knowledge sharing, and stakeholder development parts were not designed to stand alone in Dimension three due to their significance in multiple framework items. The elements of these development parts were therefore integrated into one or more of the framework’s items as supporters/influences of the respective concepts.

#### Dimension Three: The Ecosystem Canvas

The ecosystem canvas, discussed in this section, forms part of Dimension three and includes the newly termed ecosystem levels, namely, the external environment, the organization, and the stakeholders. These ecosystem levels form subcategories to three categories, namely the input, strategic priorities, and activities, and output. These categories and subcategories are discussed in detail in the sections to follow.

**FIGURE 11:**
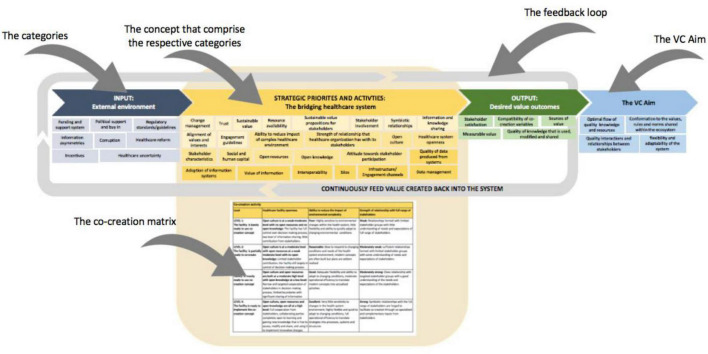
Part one of the ecosystem canvas.

The layout of the Ecosystem Canvas is presented in Figure 11. The canvas is firstly presented in the format of a feedback loop to illustrate the structure of the value creation system, which consists of the most notable concepts from literature that need to be considered when creating value centered around information systems in a healthcare ecosystem. This part of the canvas is structured in this manner to encourage and support the continuous growth, development, and improvement of a healthcare system.

**FIGURE 12:**
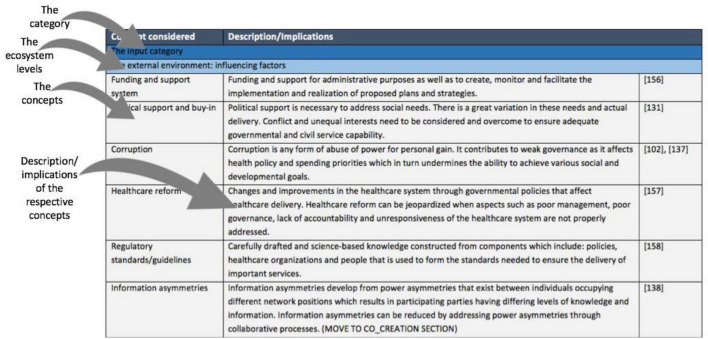
Second part of the ecosystem canvas.

The concepts included in the ecosystem canvas and their respective descriptions/implications form the second part of the canvas. An extract from this part of the canvas is presented in Figure 12. The purpose of this part is to provide the user with a better understanding of the categories and concepts that constitute the canvas. All the concepts are uniquely arranged as an appropriate way to convey the required information.

##### The Input Category

**FIGURE 13:**
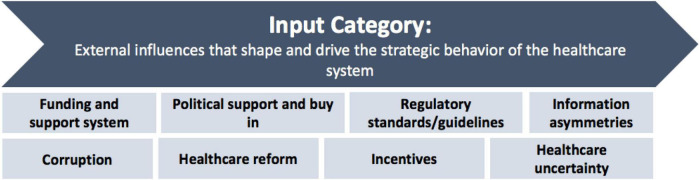
Recommended external influences consider.

In a healthcare ecosystem, the healthcare organization (which includes healthcare facilities for the sake of this explanation) does not stand alone. It consists of a network of explicit and implicit relationships that span both the internal and external environment. It is for this reason that the ecosystem canvas suggests the consideration of not only the internal factors of the organization, but also its external influences (see Figure 13). This is motivated by the need to gain a deeper understanding of the influence that these environmental factors have on the organization’s desired outcomes and to stimulate innovativeness within the healthcare organization. The healthcare organization relies on, and is greatly influenced by, changes within its external environment. These external influences govern the healthcare ecosystem and therefore shapes the structure of the healthcare organization. It is for this reason that the external environment is considered an input that drives the strategic behavior of a healthcare system, hence its placement in the input category of the ecosystem canvas. The most notable external influences from literature are included in the input category. These influences should be considered as constraints or enablers of the healthcare system’s ability to reach the desired healthcare outcomes. The input category recommends that users of the canvas consider the external influences shown in Figure 13, which span the healthcare organization’s external environment, to make informed decisions.

These external influences have an important role in the complexity of the healthcare system, as the external environment is dynamic and continuously changing. It is important to consider these influences to reduce the impact of the complexity of the external environment, to adapt faster to changes and make better decisions.

##### The Strategic Priorities and Activities Category

**FIGURE 14:**
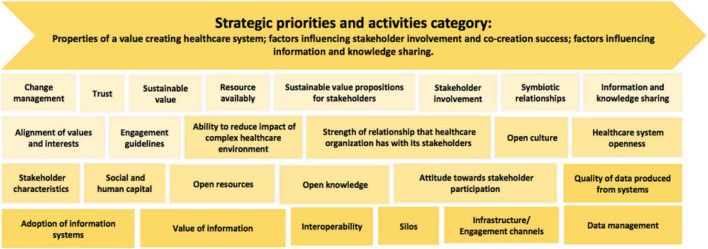
Recommended strategies and activities to consider.

Strategic priorities and activities are in place to define and redefine the way in which a healthcare system operates. This category represents concepts that were thoughtfully put together in response to the challenges within the healthcare system that affect value creation. This category is designed to equip users to effectively engage and support one another during the value creation process. Its focus is to highlight recommended concepts to consider regarding, first, the properties of a value creating healthcare system; second, factors influencing stakeholder involvement and co-creation success; and, third, factors influencing information and knowledge sharing. These concepts are presented in Figure 14.

The properties of a value creating healthcare system provide a general understanding of how a healthcare ecosystem could function, as well as what needs to be considered to cultivate a collaborative environment. This type of environment is essential for bringing together multiple stakeholders with varying stakes in the healthcare system for the purpose of jointly developing sustainable solutions, while still providing a platform for value creation. Each property in the healthcare system has a role in the value creation process. Lack of attention and recognition of this role can result in the structure and initiatives of the healthcare system becoming inadequate. It is therefore important to have a holistic view of all the parts of the system to understand the interrelatedness of the components and to gain a deeper understanding of where and how value emerges. The idea is to foster learning networks in healthcare systems that encourage and support continuous improvement of practices and institutional development.

The healthcare system provides a space for learning and knowledge sharing, co-construction of new innovations, and value creation. It is important for collaborative networks to exist in such a space, as they play a vital role in ensuring that these objectives are met through the continuous use of co-creation practices. Deliberate implementation of the latter is necessary, as the degree of advancement of the healthcare organization within its ecosystem heavily depends on these co-creation practices; further, they are necessary for a competitive advantage and to drive innovation. A key enabler of co-creation is stakeholder involvement, since co-creation is a function of stakeholder interactions. Exploring co-creation through the engagement of multiple stakeholder groups is essential for the improvement of healthcare services. Successful co-creation requires stakeholders to interact and build strong relationships through the exchange and integration of resources in the healthcare system; hence, the ecosystem canvas focuses on the factors that influence stakeholder involvement. These factors can be viewed as either obstacles to, or supporters of, the process, and comprise notable factors that were identified from literature and interviews, which were considered as important to the context.

Information and knowledge sharing is essential for the survival of a healthcare organization within its ecosystem, and it is therefore, in the context of the use of information systems, another prominent concept in the ecosystem canvas. Lack of information and knowledge sharing can be detrimental and affect the success of the healthcare system. It is therefore necessary to encourage transparency within the healthcare system, where free and unrestricted information and knowledge is available for use by relevant stakeholders. The successful adoption and implementation of information systems play a larger role here. In the healthcare system, information systems have the potential to improve the quality of care received by patients and the management of healthcare costs. Furthermore, if well directed, information systems can be used to facilitate information and knowledge sharing between stakeholders for the purpose of co-producing value for the healthcare system. Literature and interview data confirm the importance of information and knowledge sharing in the success of value creation, hence its inclusion in the ecosystem canvas. Information and knowledge sharing streamlines the health system through information systems, thus creating value. This ultimately results in improved communication, effective management of healthcare practices, improved resource allocation, and efficient resource flow; all of which are essential to efficient service delivery. To harness value from information and knowledge in the healthcare system, the ecosystem canvas places emphasis on the components that facilitate the adoption, use, and management of information and knowledge to support the healthcare processes to create value. In this way, the canvas encourages the need to understand the environment that the information system functions and how it links to the success of the healthcare system.

##### The Output Category

**FIGURE 15:**
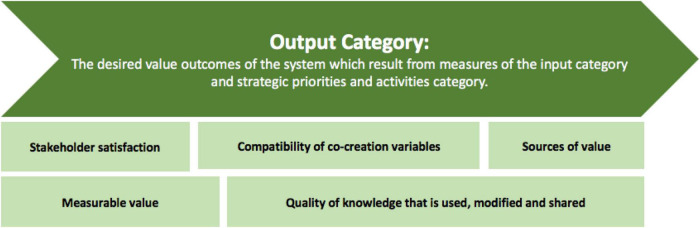
Recommended value outcomes to be consider.

Through a comprehensive and holistic view of the healthcare system, the ecosystem canvas links the preceding categories of the canvas to the output category, as they directly and indirectly affect the desired value outcomes. The structure of the canvas therefore suggests that a deeper understanding of the two preceding categories is necessary to understand the impact that they have on the desired outcomes of the healthcare system. Further, an understanding of the desired outcomes of a value creation process is important to identify areas for improvement, as this determines the necessary activities that need to be performed by the organization. The output category recommends users of the canvas to consider the factors shown in Figure 15, as they compare to the operational and strategic performance of the healthcare system.

The value outcomes included in the ecosystem canvas are important to be achieved as they encompass many of the goals inherent to healthcare such as quality, patient centeredness, and cost management. The factors in the output category were included for their role and significance.

##### The Value Creation Aim

The Value Creation (VC) aim was included in the management tool to serve as guide to track the success of the healthcare system and to drive progress. It intends to achieve this by focusing on the following four integrated objectives: (1) optimal flow of quality knowledge and resources; (2) conformation to values, rules, and norms shared within the ecosystem; (3) quality interactions and relationships between stakeholders; and (4) flexibility and adaptability of the system. These objectives are important as they recognize the fundamental principles of value creation and the role of key stakeholders that are needed to achieve systemic excellence.

The *optimal flow* of quality knowledge and resources considers how quality information and knowledge is streamlined in the healthcare system to improve communication between stakeholders, management of healthcare practices, resource allocation, and efficient resource flow. *Conformation to values, rules, and norms* shared within the ecosystem considers the governance mechanisms that are in place to support key actors in co-creating value in a manner that can advance the healthcare system. This is vital for the creation, development, health, and maintenance of the healthcare ecosystem. *Quality interactions and relationships* between stakeholders is an essential dimension that is necessary in realizing value creation. These interactions and relationships are expressed through the functional characteristics of each stakeholder, as well as through their responsibility in the ecosystem. *Flexibility and adaptability* of the system refers to the systems’ ability to adapt to changes or disturbances in the healthcare ecosystem. The healthcare system needs to have the ability to either return to its original state of equilibrium, or adapt to a new equilibrium.

## Conclusion

The implementation of the management tool and framework needs to occur through the activities of the healthcare system’s business/operating model, which is central to the value creation process. The business model considers all the resources, capital, and relationships in an integrated manner, and turns these valuable resources into desired outputs. Implementation of the management tool is proposed to be done in a three-stage process, managed by project management practices, to ensure that the appropriate knowledge, skills, and resources are used to achieve the objectives. This is essential as the healthcare ecosystem is complex in nature. The three-stage process should include: (1) planning; (2) execution; and (3) evaluation. During the planning stage, the healthcare organization will need to define their goals by describing how they intend on moving from the system’s current state to their envisioned state. The implementation of the management tool takes place during the execution stage. The researcher suggests the use of change management tools to assist in managing the launch of the value creation management tool to minimize the impact on the various stakeholder groups and the healthcare organization. Finally, the implementation process can be evaluated to monitor the use of the management tool in the healthcare organization and to ensure the transition to newly implemented practices is seamless. To this end, is important to consider the people, processes, and culture of the healthcare organization.

### Contribution to the Literature

The framework and management tool conceptualizes and characterizes important strategic features of a value creation system from a holistic perspective. The framework comprises interdependent components that were identified from existing literature [see section “Literature Review (Part 1 – Relevance and Rigor Cycle)”] and synthesized and organized into a practical management tool. This work contributes to a burgeoning literature on better understanding and managing value creation from an ecosystem perspective (Matthies et al., 2016; Barile et al., 2020; Botti and Monda, 2020; Autio, 2021).

The content of the framework is intended to stimulate thought and provide users with an understanding of how elements within a healthcare ecosystem can influence the value creation process. The tool offers a novel course of action that can be taken to create sustainable value in a healthcare system by considering: (1) important input factors and external influences; (2) strategic activities that can be performed; and (3) the desired outcomes that may be achieved. The desired value outcomes highlighted in the framework, together with co-creation matrix, the VC Aim and structure of the framework, inform the continuous improvement initiatives within a healthcare system to drive efficiencies through the use of information systems. Furthermore, the structure of the framework encourages the need to feed value created within a healthcare system back into the system to drive progress. The contribution made in this article is evaluated in the South African context where a proven gap exists for the evaluation of practical solutions (Hlongwane and Grobbelaar, 2020).

### Managerial Implications

The framework and management tool comprises interdependent components that were uniquely organized to stimulate thought and provide users with an understanding of how elements within a healthcare ecosystem can influence the value creation process. The tool offers a course of action that can be taken to create sustainable value in a healthcare system by considering: (1) important input factors and external influences; (2) strategic activities that can be performed; and (3) the desired outcomes that may be achieved. The framework informs the continuous improvement initiatives within a healthcare system to drive efficiencies using information systems. Furthermore, the structure of the framework encourages the need to feed the value created within a healthcare system back into the system to drive progress.

When using the management tool, it is important to consider the following:

1. The management tool provides a broad conceptualization of value creation in healthcare. Users need to contextualize the management tool to align with the intended scope.
2. Though an ecosystem perspective was adopted, the management tool does not account for every possible aspect that is associated with value creation in the context of health information systems.
3. The management tool is one that is conceptual and therefore a sufficient understanding of the healthcare environment prior to its use is essential. This is necessary to utilize the framework in a way that ensures that the best solutions are developed in an efficient manner.
4. Although the ecosystem canvas presents a simplistic value creation process, the value creation system considers multiple variables that are intrinsically complex. Therefore, iteration between categories may be necessary to ensure that each is addressed comprehensively. The illustration of how and where the iteration may take place falls beyond the scope of the research. This may be further investigated in future research.
5. The management tool was not designed to predict an outcome. It was designed as a conceptual framework with the intention of only improving our understanding of the phenomena in question. The use of the tool serves to inform the user’s interpretation of the phenomena in a specific context.

### Limitations and Further Research

A critical reflection on the literature reviews, evaluation processes, and final tool revealed several aspects that were not pursued within the scope of the study; these may be explored during future research:

1. The literature review was only conducted by one researcher, leaving the characterization and interpretation of the findings subject to reviewer bias.
2. The semi-structured interviews were limited in number. Therefore, more interviews with individuals from varying disciplines could have led to more complete results.
3. Only one researcher analyzed the interview data, which may have introduced bias during the coding cycle process and use of the analytical lenses.
4. The framework ranking exercise was limited in the number of participants and diversity of their backgrounds. More participants from varying disciplines and backgrounds could have led to better and possibly different results.
5. The interpretation of the findings from the evaluation processes depended on the researcher’s understanding, and could have been subject to bias.
6. The framework comprises several concepts and elements that were not investigated in-depth.

7. The framework only includes the most notable concepts from literature to comprise the input category, the strategic priorities and activities category, and the output category. The consideration of additional concepts may influence the framework.
8. The framework does not show the relative importance and actual weight of each concept regarding value and its creation.
9. The framework was developed to be a s general as possible; it does not account for all the complex and diverse aspects of a healthcare system.
10. The framework needs to continuously evolve to remain usable within complex healthcare ecosystems supported by information systems.

## Data Availability Statement

The datasets presented in this article are not readily available because in-depth experts interviews were conducted and was analyzed through qualitative analysis techniques. Requests to access the datasets should be directed to SG, ssgrobbelaar@sun.ac.za.

## Ethics Statement

The studies involving human participants were reviewed and approved by Research and Ethics Committee (REC) of Stellenbosch University; NG-2020-16817. The patients/participants provided their written informed consent to participate in this study.

## Author Contributions

Both authors actively and significantly participated in the drafting of the article and approved the submitted version.

## Conflict of Interest

The authors declare that the research was conducted in the absence of any commercial or financial relationships that could be construed as a potential conflict of interest.

## Publisher’s Note

All claims expressed in this article are solely those of the authors and do not necessarily represent those of their affiliated organizations, or those of the publisher, the editors and the reviewers. Any product that may be evaluated in this article, or claim that may be made by its manufacturer, is not guaranteed or endorsed by the publisher.
