# Supplementary material for: A Practical Framework for Value Creation in Health Information Systems From an Ecosystem Perspective: Evaluated in the South African Context
Source: Front Psychol. 2022 Jun 2;13:637883. doi: 10.3389/fpsyg.2022.637883 (PMC9201401; doi:10.3389/fpsyg.2022.637883)
Supplement: Supplementary file 1 [file Data_Sheet_1.docx]

# Supplementary materials

## Primary studies included in the literature review

Table 2: Primary sources from literature review

| Number | Author(s) | Paper title | Reference: |
| --- | --- | --- | --- |
| 1 | Osório, A Luis;  Afsarmanesh, Hamideh;  Camarinha-Matos, Luis M. | Open Services ecosystem supporting collaborative networks | (Rabionet, 2011) |
| 2 | Serbanati, Luca Dan; Ricci, Fabrizio L; Mercurio, Gregorio; Vasilateanu, Andrei | Steps towards a digital health ecosystem | (Osório *et al.*, 2010) |
| 3 | Bezák, Peter; Bezáková, Magdaléna | Landscape capacity for ecosystem services provision based on expert knowledge and public Perception (case study from the north-west Slovakia) | (Serbanati *et al.*, 2011) |
| 4 | Barrett, Michael;  Davidson, Elizabeth;  Vargo, Stephen | Service innovation in the digital age: Key contributions and future directions | (Bezák and Bezáková, 2014) |
| 5 | Sim, Sarah;  King, Henry;  Price, Edward | The role of science in shaping sustainable business: Unilever case study | (Barrett *et al.*, 2015) |
| 6 | Abellá-garcía, Alberto;  Ortiz, Marta;  De-pablos-heredero, Carmen | The Ecosystem of Services Around Smart Cities: An Exploratory Analysis | (Sim *et al.*, 2016) |
| 7 | Kharrazi, Ali;  Fath, Brian D;  Katzmair, Harald | Advancing empirical approaches to the concept of resilience: A critical examination of panarchy, ecological information, and statistical evidence | (Abellá-garcía *et al.*, 2015) |
| 8 | Aulkemeier, Fabian;  Paramartha, Mohammad Anggasta;  Iacob, Maria-eugenia;  Hillegersberg, Jos | A pluggable service platform architecture for e-commerce | (Kharrazi *et al.*, 2016) |
| 9 | Zhang, Meng;  Gable, Guy;  Rai, Arun | Toward principles of construct clarity: Exploring the usefulness of facet theory in guiding conceptualization | (Aulkemeier *et al.*, 2016) |
| 10 | Grêt-regamey, Adrienne;  Altwegg, Jürg;  Sirén, Elina A;  Strien, Maarten J Van;  Weibel, Bettina | Landscape and Urban Planning Integrating ecosystem services into spatial planning — A spatial decision support tool | (Zhang *et al.*, 2016) |
| 11 | Adler-Milstein, Julia;  Embi, Peter J;  Middleton, Blackford;  Sarkar, Indra Neil;  Smith, Jeff | Crossing the health IT chasm: Considerations and policy recommendations to overcome current challenges and enable value-based care | (Grêt-regamey *et al.*, 2017) |
| 12 | Park, Sohyun | A preliminary study on connectivity and perceived values of community green spaces | (Adler-Milstein *et al.*, 2017) |
| 13 | Liu, Delin;  Hao, Shilong | Ecosystem health assessment at county-scale using the pressure-state-response framework on the loess plateau | (Park, 2017) |
| 14 | Pappas, Ilias O;  Mikalef, Patrick;  Giannakos, Michail;  Krogstie, John;  Lekakos, George | Big data and business analytics ecosystems: Paving the way towards digital transformation and sustainable societies | (Liu and Hao, 2017) |
| 15 | Tarafdar, Monideepa;  Tanriverdi, Hüseyin | Impact of the information technology unit on information technology-embedded product innovation | (Pappas *et al.*, 2018) |
| 16 | Schiza, Eirini;  Kyprianou, Theodoros;  Petkov, Nicolai;  Schizas, Christos; | Proposal for an eHealth Based Ecosystem Serving National Healthcare | (Tarafdar *et al.*, 2018) |
| 17 | Joda, Tim;  Waltimo, Tuomas;  Probst-Hensch, Nicole;  Pauli-Magnus, Christiane;  Zitzmann, Nicola | Health Data in Dentistry: An Attempt to Master the Digital Challenge | (Schiza *et al.*, 2019) |
| 18 | Heim, Irina;  Kalyuzhnova, Yelena;  Li, Weizi;  Liu, Kecheng | Value co-creation between foreign firms and indigenous small- and medium-sized enterprises (SMEs) in Kazakhstan's oil and gas industry: The role of information technology spillovers | (Joda *et al.*, 2019) |
| 19 | Müller, Matthias;  Vorraber, Wolfgang;  Slany, Wolfgang | Open principles in new business models for information systems | (Heim *et al.*, 2019) |
| 21 | Pesce, Danilo  Neirotti, Paolo  Paolucci, Emilio | When culture meets digital platforms: value creation and stakeholders’ alignment in big data use | (Pesce *et al.*, 2019) |
| 22 | Talmar, Madis  Walrave, Bob  Podoynitsyna, Ksenia S.  Holmström, Jan  Romme, A. Georges L. | Mapping, analyzing, and designing innovation ecosystems: The Ecosystem Pie Model | (Talmar *et al.*, 2020) |
| 23 | Ferreira, Luís Miguel  Menezes, João Carlos | How costumers' way of life influences the value co-creation | (Ferreira and Menezes, 2020) |
| 24 | Nöjd, Sture  Trischler, Jessica Westman  Otterbring, Tobias  Andersson, Pernille K.  Wästlund, Erik | Bridging the valuescape with digital technology: A mixed methods study on customers’ value creation process in the physical retail space | (Nöjd *et al.*, 2020) |
| 25 | Dell'Era, Claudio  Di Minin, Alberto  Ferrigno, Giulio  Frattini, Federico  Landoni, Paolo  Verganti, Roberto | Value capture in open innovation processes with radical circles: A qualitative analysis of firms’ collaborations with Slow Food, Memphis, and Free Software Foundation | (Dell’Era *et al.*, 2020) |

## Important concepts

Table 4: Important concepts identified from literature

| Search term: | Category: | Key concept: | Description: | Reference(s) |
| --- | --- | --- | --- | --- |
| Information systems | Interoperability | Integration | Integration of dissimilar systems to improve wellness, quality, safety, and cost-effectiveness. | (Osório *et al.*, 2010), (Aulkemeier *et al.*, 2016), (Schiza *et al.*, 2019), (Hardyman *et al.*, 2015) |
|  |  | Collaborative networks | An interaction across local/ wide area networks regardless of physical and operating systems. | (Schiza *et al.*, 2019), (Serbanati *et al.*, 2011), (Zhang *et al.*, 2016), (Tarafdar *et al.*, 2018), (Osório *et al.*, 2010) |
|  |  | Authority/  authorisation | To create a regulating body that has legal powers to enforce standards in services. | (Schiza *et al.*, 2019) |
|  | Stakeholder-related | Value co-creation | The mediation of technology platforms in stakeholder interactions leads to the creation of value. | (Heim *et al.*, 2019), (Barrett *et al.*, 2015) , (Müller and Vorraber, 2019), (Zhang *et al.*, 2016), (Hardyman *et al.*, 2015) |
|  |  | Usefulness | Supports decision-making procedures to ensure delivery of service at point of need. | (Schiza *et al.*, 2019), (Serbanati *et al.*, 2011), (Joda *et al.*, 2019) |
|  |  | Acceptance/  Adoption | Acceptance of information systems, and therefore its success, largely depends on the mindset and culture of the stakeholders. | (Schiza *et al.*, 2019), (Serbanati *et al.*, 2011) |
|  |  | User information satisfaction | The extent to which the user believes that the information system can meet their information requirements. | (Zhang *et al.*, 2016) |
|  | Pluggability | Quality | Quality standards that are a reflection of the external quality criteria for IT services. These standards are equivalent to reliability, efficiency, and/or maintainability. | (Aulkemeier *et al.*, 2016) |
|  | Sustainability | Management  information systems | Fosters compliance with documentation standards by ensuring that data are structured, accurate, relevant and complete. | (Joda *et al.*, 2019), (Tarafdar *et al.*, 2018), (Schiza *et al.*, 2019) |
|  |  | Service innovation | Drives economic development and leads to greater efficiency and cost reduction. | (Barrett *et al.*, 2015) |
|  |  | innovation management | Leads to the development of innovative digital data-based designs that transform businesses. | (Pappas *et al.*, 2018) |
| Ecosystem | Resilience | Adaptability | A system that can either return to its original state of equilibrium or a system that adapts to a new equilibrium. | (Kharrazi *et al.*, 2016) |
|  |  | Actors | Diverse actors that function in different stages when a disturbance in the system occurs. | (Kharrazi *et al.*, 2016) |
|  |  | Duality | A system may be resilient in a condition that can be viewed as either positive or negative. | (Kharrazi *et al.*, 2016) |
|  | Functioning | Actors | A multitude of agents that are interconnected and that integrate resources to co-create value in the ecosystem. | (Schiza *et al.*, 2019), (Serbanati *et al.*, 2011), (Barrett *et al.*, 2015) |
|  |  | Collaboration, cooperation, coopetition | Creates opportunities to address stakeholder needs by developing innovative designs. | (Pappas *et al.*, 2018), (Osório *et al.*, 2010), (Müller *et al.*, 2019) |
|  |  | Innovation | Evolving the way in which ecosystem actors interact, cooperate, and collaborate. | (Pappas *et al.*, 2018), (Müller *et al.*, 2019) |
|  |  | Value co-creation | The creation of value through interaction and coproduction of knowledge between different actors in a multiple-stakeholder environment. | (Heim *et al.*, 2019), (Barrett *et al.*, 2015), (Müller *et al.*, 2019) |
|  | Ecology/natural life | Biological community | The interaction of living organisms with their nonliving environment. | (Pappas *et al.*, 2018), (Serbanati *et al.*, 2011) |
| Value | Concepts to consider | Data-driven culture | Extracting data that have purpose and meaning in order to give actionable insight to allow decision makers to base their decisions on insight instead of instinct. | (Pappas *et al.*, 2018), (Joda *et al.*, 2019) |
|  |  | Dynamic capabilities | Actively integrating stakeholders with varying needs and capabilities to increase value. | (Pappas *et al.*, 2018), (Adler-Milstein *et al.*, 2017) |
|  |  | Learning | Continuously monitoring and learning about the evolving perceptions and needs of stakeholders in order to create value. | (Tarafdar *et al.*, 2018) |
|  |  | Communities | Create value through the collaboration of various actors that are bonded together by their competences, relationships, information, and shared vision. | (Barrett *et al.*, 2015), (Müller *et al.*, 2019) |

## Conceptual inventory framework

Table 5: The preliminary conceptual framework

| **Dimension** | **Considerations** | **Description** | **Reference** |
| --- | --- | --- | --- |
| The external environment | Funding and support system | - Administrative purposes. - To create, monitor, and facilitate the implementation and realisation of proposed plans and strategies. | (Medema *et al.*, 2017) |
|  | Political support and buy-in | - Address social needs. - Address conflict and unequal interests. | (Medema *et al.*, 2017) |
|  | Regulatory standards/guidelines | - Consider policies, healthcare organisations, and people. - Needed to ensure the delivery of important services. | (Iansiti and Levien, 2017), (Whiteside, 2014) |
|  | Incentives | - Reward structures to promote better performance. | (Primmer *et al.*, 2015) |
|  | Healthcare uncertainty | - Affects the performance and commitment of ecosystem actors. | (Medema *et al.*, 2017) |
|  | Information asymmetries | - Differing levels of knowledge and information between individuals occupying different network positions. | (Geersbro and Ritter, 2010) |
|  | Initial trust levels | - Provide insight on the state of the relationships between different stakeholders within the collaborative network. | (Medema *et al.*, 2017) |
| **Dimension** | **Considerations** | **Description** | **Reference** |
| The healthcare facility/organisation | Stakeholder involvement | - The involvement of crucial and diverse stakeholders is essential for successful value creation. | (Medema *et al.*, 2017) |
|  | Engagement guidelines | - Provide a basic structure to manage and orchestrate the way in which communication takes place between different stakeholder groups. | (Medema *et al.*, 2017) |
|  | Alignment of values and interests | - Differing values and interests between stakeholder groups pose a threat to their relationships and therefore need to be aligned. | (Hein et al. 2016) |
|  | Information and knowledge sharing | - Involves distribution of information for systems, organisations, and people. | (Geersbro *et al.*, 2010) |
|  | Resource availability | - Resources are necessary requisites to build the facility/organisation’s value creation capacity. | (Lotfi *et al.*, 2013) |
|  | Interoperability | - Allows for meaningful and reliable use of information through the use of information systems. - Encourages data quality and consistency. | (Medema *et al.*, 2017) |
| **Dimension** | **Considerations** | **Description** | **Reference** |
| Co-creation | Open culture | - Stimulates the facility/organisation’s readiness and preparedness to co-create value. - Plays a strategic role in how the facility/organisation adapts to changes. | (Sachdeva and Bhalla, 2012; Panetto *et al.*, 2016) |
|  | Open resources | - Open resources are important as they encourage the network of resources and the integration of activities. | (Adamik *et al.*, 2018) |
|  | Open knowledge | - Occurs when everyone has free access to knowledge and are able to use, modify, and share it. | (Adamik *et al.*, 2018), (Thomas *et al.*, 2012) |
|  | Healthcare facility openness | - Refers to the actions within the primary healthcare facility that open the facility up to its environment through transparency, access to knowledge and information, and collaboration. | (Adamik *et al.*, 2018), (Thomas *et al.*, 2012) |
|  | The ability to reduce the impact of the complexity of the health facility/organisation environment | - Necessary to increase the certainty of its activities and ability to exploit opportunities emerging in the environment. | (Adamik *et al.*, 2018), (Gibson, 2012) |
|  | The strength of the relationship that the facility has with its stakeholders | - Strong relationships with stakeholders can reduce the impact of the complex environment. | (Adamik *et al.*, 2018), (Kharrazi *et al.*, 2016) |
| **Dimension** | **Considerations** | **Description** | **Reference** |
| Stakeholders | Readiness and ability to participate in value co-creation | - Readiness of stakeholders to interact with one another through direct interactions to co-create value. | (Adamik *et al.*, 2018) |
|  | Symbiotic relationships | - Understanding the interrelationships and dependencies that arise between participants is important in order to understand how value is created and delivered within the healthcare ecosystem. | (Grönroos *et al.*, 2013), (Thomas *et al.*, 2012) |
| **Dimension** | **Considerations** | **Description** | **Reference** |
| Value outcomes | Compatibility of co-creation variables | - Value co-creation is driven by the relationships between participants, the environment, and the healthcare facility itself. These variables need to be compatible to successfully co-create value. | (Ekanayake *et al.*, 2017) |
|  | Measured value | - The value created can serve as an indicator of the success of value co-creation activities. - Measuring the value allows for action to be taken for further improvement. | (Adamik *et al.*, 2018) |
|  | Sources of value | - Identifying and understanding the different sources of value help to understand value logic in an ecosystem and how value is and can be co-created in the ecosystem. | (Adamik *et al.*, 2018) |
|  | Quality of knowledge that is used, modified, and shared | - Quality knowledge is a necessary outcome of value creation as it allows for disparate elements of knowledge to be identified and used in cohesive ways. | (Thomas *et al.*, 2012) |

## Interview discussion guidelines for semi-structured interviews

***Introduction and background***

- Researcher will introduce herself
- A background on the research will be presented to the interviewee using PowerPoint slides
- This will lead to the researcher discussing why the interviewee is relevant to validate the research

***Permission and consent to participate***

- The researcher will discuss the confidentiality and terms of engagement prior to the commencement of the interview
- Interviewee will then need to sign the consent form

***Interview discussion***

**Introductory questions**

1. With regards to the use of information systems in healthcare, that you have experience with, what is the desired aim that is to be achieved? How is value intended to emerge from the use of information systems?

**Establish the need for a value creation system framework**

1. How important is it to create value through health information systems?
2. How many resources and effort go into creating value in healthcare?
3. Are there standard processes or protocols followed during the value creation processes in healthcare?
4. Are there existing frameworks or guidelines that are currently being used to create value through health information systems?
   1. If so, to what extent are they used?
   2. Do you think these frameworks or guidelines are effective in creating value in the healthcare system?

**Evaluate the preliminary framework**

1. What strategies are followed when creating value in healthcare by using information systems?
2. What should be considered regarding the functioning of a health information system when it comes to value creation?

Various concepts from literature emerged regarding the value creation of information systems in primary healthcare. These concepts formed the building blocks that support the creation and success of the operations of a value creation system. I want to focus on these now.

1. Has X been considered during the design, development, and implementation of the value creation system?
2. If so, is it something you would regard as important?
3. How has it been applied?
4. Please mention some ways in which X has been realized?
5. Have there been any instances when the desired goals of X may not have been realized?
6. What influence does X have in the success of the value creation system?
7. What should be considered regarding X of the health system?
8. How can X evolve within its ecosystem?
9. Are there any concepts that you would regard as important in the context of value creation, but are often overlooked?
10. It is possible that some concepts may have been overlooked in the research study. Are there any that you would consider to be important but are not present in the framework?
11. Are there any relations between the concepts that you would deem important but have often been overlooked?
12. Are there any relationships between concepts in the framework that you would deem important that may have been overlooked in the research study?

X denotes a related concept may be inserted to complete the question.

**Conclusion and thanks**

1. Do you have any questions that you would like to ask regarding the research study?
2. Would you be willing to do a follow-up survey on the adjusted framework?

Thank you for your time.

## The framework ranking interview process


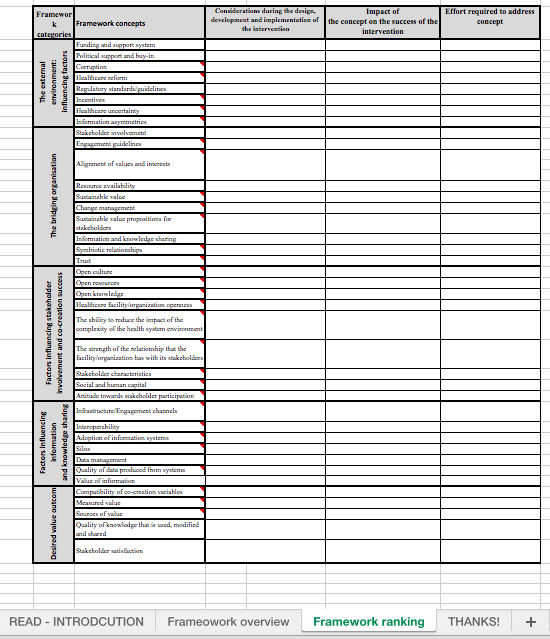
Table 6: The framework ranking sheet

## Evaluated management tool: Dimension one: The Pre-use canvas


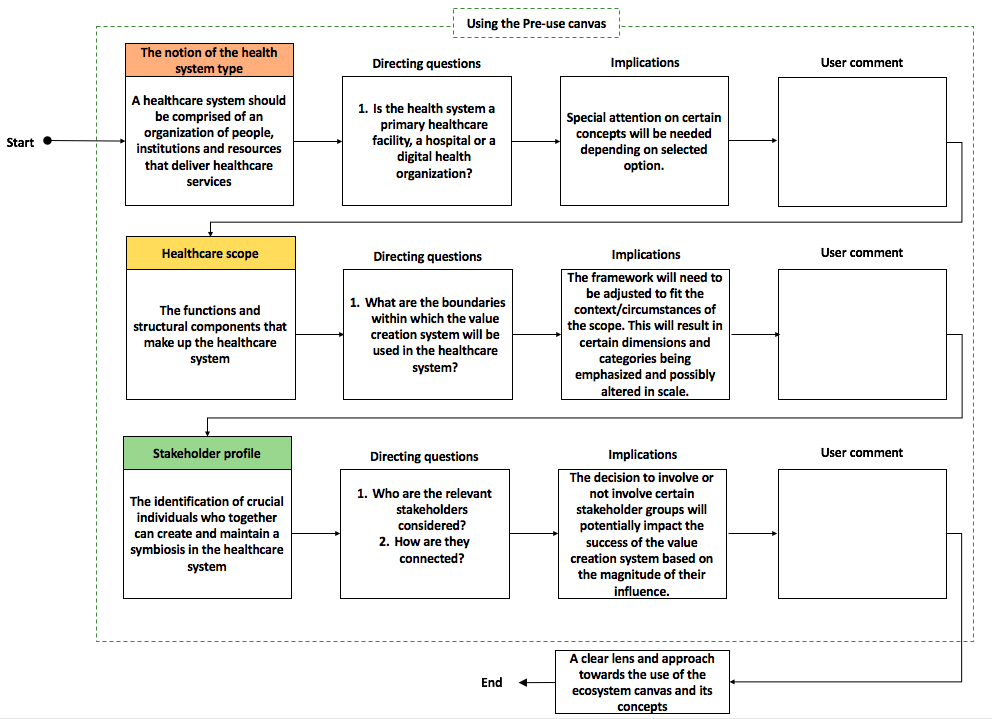


## Evaluated management tool: Dimension two: The tool guideline


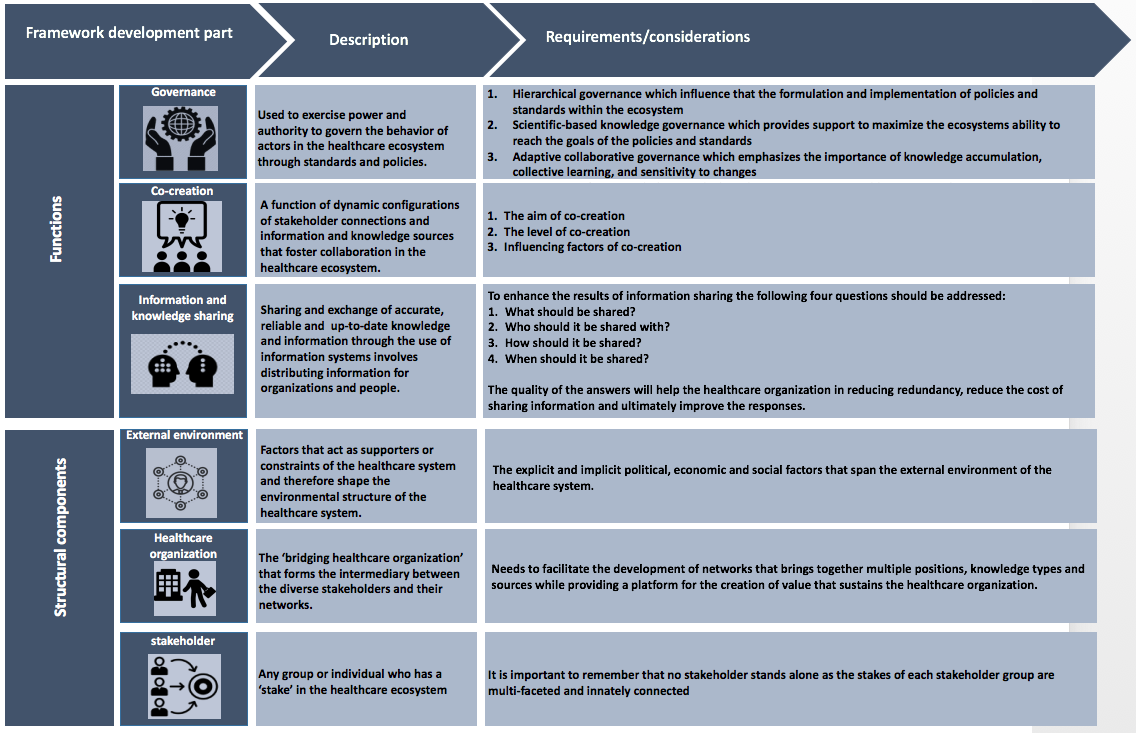


## Evaluated management tool: Dimension three: The ecosystem canvas

Ecosystem canvas Part one: The structure


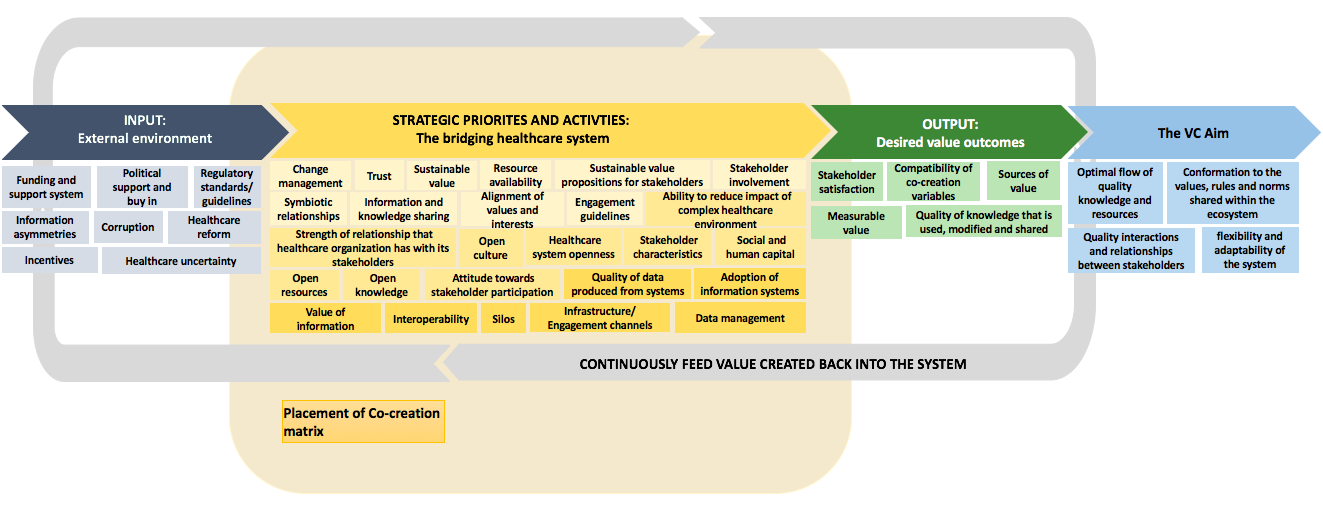


### Evaluated co-creation Matrix

| **Co-creation activity** | | | |
| --- | --- | --- | --- |
| **Level of co-creation** | **Healthcare facility openness** | **Ability to reduce the impact of environmental complexity** | **Strength of relationship with full range of stakeholders** |
| **LEVEL 1:** | **Open culture is at a weak-moderate level with no open resources and no open knowledge:** The facility has full control over decision-making process, low level of information sharing, little contribution from stakeholders. | **Poor:** Highly sensitive to environmental changes within the health system, little flexibility and ability to quickly adapt to changing environmental conditions. | **Weak:** Relationships formed with limited stakeholder groups with little understanding of needs and expectations of full range of stakeholders. |
| **LEVEL 2:** | **Open culture is at a moderate level with open resources at a weak moderate level with no open knowledge:** Limited stakeholder contribution, the facility still largely in control of decision-making process. | **Reasonable:** Slow to respond to changing conditions and needs of the health system environment; modern concepts are often built but plans are seldom realized. | **Moderately weak:** sufficient relationships formed with limited stakeholder groups with some understanding of needs and expectations of stakeholders. |
| **LEVEL 3:** | **Open culture and open resources are both at a moderate high level with open knowledge at a low level:** Narrow and targeted cooperation of stakeholders in decision-making process, limited boundaries with significant sharing of information | **Good:** Adequate flexibility and ability to adapt to changing conditions, moderate operational efficiency to translate modern concepts into actualized activities. | **Moderately strong:** Close relationship with targeted stakeholder groups with a good understanding of the needs and expectations of the stakeholders. |
| **LEVEL 4:** | **Open culture, open resources and open knowledge are all at a high level:** Full cooperation from stakeholders; collaborating parties completely open to learning and gaining new knowledge that is free to access, modify, and share, and using it to implement innovative changes. | **Excellent:** Very little sensitivity to changes in the health system environment; highly flexible and quick to adapt to changing conditions, full operational efficiency to translate strategies into processes, systems, and structures | **Strong:** Symbiotic relationships with the full range of stakeholders are forged to facilitate co-creation through co-specialized and complementary inputs from stakeholders |

### Ecosystem Canvas part two: Conceptual framework

| **Framework elements** | **Description/Implications** |
| --- | --- |
| **The input category:** | |
| **The external environment: influencing factors** | |
| Funding and support system | - Funding and support to create, monitor, and facilitate the implementation and realization of proposed plans and strategies. |
| Political support and buy-in | - Conflict and unequal interests create great variation in social needs and actual delivery. Need to be considered and overcome to ensure adequate governmental and civil service capability. - The existence of a political-to-expertise imbalance that results from leadership positions being taken up by people with political affiliations as opposed to individuals with appropriate qualifications. - The influence of the political-to-expertise imbalance on decisions made by the government. |
| Regulatory standards/guidelines | - Carefully drafted, science-based knowledge constructed from components, which include policies, healthcare organizations, and people. - Standards are needed to direct the delivery of important services. |
| Information asymmetries | - Information asymmetries resulting from power asymmetries that exist between individuals occupying different network positions. - Participating parties having differing levels of knowledge and information. |
| Corruption | - Corruption is any form of abuse of power for personal gain. It contributes to weak governance as it affects health policy and spending priorities. - Undermines the ability to achieve various social and developmental goals. |
| Healthcare reform | - Changes and improvements in the healthcare system through governmental policies that affect the way in which healthcare is delivered. - Healthcare reform can be jeopardized when aspects such as poor management, poor governance, lack of accountability, and unresponsiveness of the healthcare system are not properly addressed. |
| Incentives | - Reward structures that are put in place to promote better performance. - May be used to encourage participation with stakeholders whose interests and values may differ. |
| Healthcare uncertainty | - Uncertainty can negatively affect the performance and commitment of actors within the healthcare ecosystem due to the inability to gain full insight on all aspects of the ecosystem. |
| **Framework elements** | **Description/Implications** |
| **The strategic priorities and activities category:** | |
| **Properties of a value creating healthcare system** | |
| Change management | - Actions taken to ensure the smooth transition from the current state of the healthcare system to the desired future state. - Key steps for successful change management include: (1) assessing the readiness to change; (2) establishing a sense of urgency; (3) assembling a steering team; (4) developing an implementation plan; (5) executing the pilot; (6) disseminating change; and (7) anchoring the change. |
| Trust | - Trust built between participating individuals from previous personal and informal interactions. - Trust levels provide insight into the state of relationships between different stakeholders within the collaborative network. - Necessary to sustain relationships between stakeholders. |
| Sustainable value | - Sustainable value creation is multifaceted, involving economic, social, and environmental concerns. - The creation of sustainable value involves carrying out healthcare services that sustain the healthcare system through the efficient use of resources. |
| Resource availability | - Necessary requisites for systems to build their value creation capacity. - Limited resources can hinder the value creation process and can be linked to low frequency of communication and interactions between stakeholders. |
| Sustainable value propositions for stakeholders | - The development of sustainable benefits, which can include social, economic, and environmental benefits, for stakeholders. - Proactive stakeholder engagement is necessary to: (1) reduce the systems’ sensitivity to emerging social, economic, or environmental expectations; (2) ensure that value created for one stakeholder group does not result in value destroyed for other stakeholder groups; and (3) allow for the discovery of value sources and opportunities. |
| Stakeholder involvement | - The involvement of crucial and diverse stakeholders is essential for successful value creation. - It allows for the healthcare system to see its role and function from a broader perspective. - Diverse approaches should be used to successfully bring together different stakeholder groups based on the vision, goals, and strategies of the facility/organization. If wrongly or ineffectively used, value destruction can take place, or in the best case there may be no significant impact.   Lack of involvement of end users in the decision-making process can greatly affect the value that is created. It is important to identify opportunities to involve end users. |
| **Framework elements** | **Description/Implications** |
| Symbiotic relationships | - Value logic is supported by the symbiotic relationship formed between participants. - It is vital to understand these interrelationships and dependencies that arise between participants to understand how value is created and delivered within the healthcare ecosystem. |
| Information and knowledge sharing | - More conceivable when facilitated by information systems. - Relevant stakeholders need to be willing to participate in information sharing activities. - The lack of information and knowledge sharing results in the ineffective coordination of actions and entities in the healthcare system |
| Alignment of values and interests | - Differing values between stakeholder groups pose a threat to the relationship between the stakeholders. - The failure to identify and properly align interests and values can severely damage the performance of the facility/organization. |
| Engagement guidelines | - Engagement guidelines provide a basic structure to manage and orchestrate the way in which communication takes place between different stakeholder groups. - There must be clear principles of dialogue to guide the interactions between stakeholders. There must also be an understanding between stakeholders regarding their roles and responsibilities. |
| **Factors influencing stakeholder involvement and co-creation success** | |
| The ability to reduce the impact of the complexity of the health system environment | - The complexity of the health system’s environment refers to the interdependent and interconnected entities that support the healthcare system. These include: stakeholders, technology systems, and the facility/organization structure. - The healthcare system needs to be able to reduce the impact of the complexity of its environment to increase the certainty of its activities and ability to exploit opportunities emerging in the environment. |
| The strength of the relationship that the healthcare system has with its stakeholders | - Building stronger relationships with stakeholders can reduce the impact of the complexity of the environment by reducing the sensitivity of the healthcare system to changes occurring in the environment. - It allows systems to adapt faster to the changes and possibly anticipate what is to come, to make better decisions through knowledge and information sharing. |
| Open culture | - Open culture means that a healthcare system should: (1) be open to its environment; (2) promote cooperation in its environment; (3) be open to new knowledge and changes; and (3) ensure the free formation of relationships with all relevant stakeholders. - Stimulates value co-creation in the system. - It is the backbone of a healthcare system and plays a strategic role in how a healthcare system adapts to changes. |
| **Framework elements** | **Description/Implications** |
| Healthcare system openness | - Actions within the healthcare system that open the system up to its environment. These actions are rooted in an open culture, open resources, and open knowledge. - Requires transparency, access to knowledge and information, and collaboration. |
| Stakeholder characteristics | - Influence whether stakeholders are willing to participate and co-create. - Intrinsic factors such as culture, values, level of education, and language barriers affect a stakeholder’s willingness to participate. |
| Social and human capital | - Necessary for sustainable involvement of individuals with differing levels of marginalization and access to health services. - Relationships with these individuals are not homogeneous and can differ based on the characteristics and context of the individual. |
| Open resources | - Basic factors that determine the healthcare system’s success in the age of complex environments. - Encourages the network of resources and the integration of activity. |
| Open knowledge | - Results from the solid foundation that open culture and open resources build. - Occurs when everyone has free access to knowledge to be able to use, modify, and share it. |
| Attitude towards stakeholder participation | - Attitudes of healthcare officials towards stakeholder involvement affects and influences the extent to which co-creation occurs. |
| **Factors influencing information and knowledge sharing** | |
| Quality of data produced from systems | - Drives improvement of data quality, which is essential to provide better healthcare services. - Correct, reliable, and up-to-date data is critical. Its benefits include: (1) high quality care; (2) ensuring that legal requirements and professional standards are met; and (3) supporting strategic planning and management of health and social services. |
| Adoption of information systems | - Influenced by factors that include: (1) attributes such as the perceived usefulness of the information system compared to its perceived ease of use, complexity, and quality; (2) characteristics of the healthcare individuals adopting the innovation; (3) contextual factors such as top management support and social norms; and (4) task characteristics such as difficulty and newness. |
| Value of information | - Information is a carrier of value that increases the more it is used and shared (i.e., value-in-use, value-in-exchange). - Value of information increases even more when it is accurate, reliable, and up to date. |
| Interoperability | - Interoperability allows for meaningful and effective use of information that is exchanged across platforms. - Encourages data quality and consistency. - Full potential of interoperability may be realized through users’ (i.e., healthcare workers) acceptance and adoption of information systems. |
| **Framework elements** | **Description/Implications** |
| Silos | - Prevent users from obtaining certain information and knowledge. - Restricts information and knowledge sharing, which affects collaboration amongst stakeholders and decision makers. |
| Infrastructure/Engagement channels | - Technical infrastructures and decision support architectures that support various applications for the creation of value and improvement of operational efficiency. |
| Data management | - Collecting, storing, analyzing, and distributing data using a set of effective and well-designed data procedures and structures. - Essential for strategic initiatives such as: (1) improving strategic decision making; (2) facilitating the integration of stakeholders; and (3) identifying new value opportunities. |
| **The output category:** | |
| **Desired value outcomes** | |
| Stakeholder satisfaction | - Influences sustainable growth and success of the healthcare system. - A healthcare system’s relationship with its stakeholders is important to ensure the satisfaction of all relevant stakeholders. |
| Compatibility of co-creation variables | - Value co-creation is driven by the relationships between participants, the environment, and the healthcare facility itself. These variables need to be compatible to successfully co-create value. |
| Sources of value | - Necessary to understand the value logic in the ecosystem and how value is and can be co-created in the ecosystem. Sources of value include innovation, flexibility, and efficiency |
| Measured value | - The value created within the system is a measure of the success of the value creation system and can also serve as an indicator of the success of value co-creation in the system. - Aids in identifying areas within the healthcare system that need further improvement. |
| Quality of knowledge that is used, modified, and shared | - Using, modifying, and sharing quality knowledge accelerates innovation within the ecosystem. - Quality knowledge is a necessary outcome of value creation as it allows for disparate elements of knowledge to be identified and used in cohesive ways. |
